# CdWRKY2‐mediated sucrose biosynthesis and CBF‐signalling pathways coordinately contribute to cold tolerance in bermudagrass

**DOI:** 10.1111/pbi.13745

**Published:** 2021-11-23

**Authors:** Xuebing Huang, Liwen Cao, Jibiao Fan, Guangjing Ma, Liang Chen

**Affiliations:** ^1^ CAS Key Laboratory of Plant Germplasm Enhancement and Specialty Agriculture Wuhan Botanical Garden The Innovative Academy of Seed Design Chinese Academy of Sciences Wuhan China; ^2^ Center of Economic Botany Core Botanical Gardens Chinese Academy of Sciences Wuhan China; ^3^ University of Chinese Academy of Sciences Beijing China; ^4^ College of Animal Science and Technology Yangzhou University Yangzhou China

**Keywords:** Bermudagrass, *CdWRKY2*, *CdSPS1*, *CdCBF1*, cold stress, sucrose synthesis, transgenic hairy root

## Abstract

Bermudagrass (*Cynodon dactylon*) is one of the most widely cultivated warm‐season turfgrass species around the world. Cold stress has been a key environmental factor that adversely affects the growth, development, and geographical distribution of bermudagrass; however, the underlying mechanism of bermudagrass responsive to cold stress remains largely unexplored. Here, we identified a cold‐induced WRKY transcription factor CdWRKY2 from bermudagrass and demonstrated its function in cold stress response. Overexpression of *CdWRKY2* enhanced cold tolerance in transgenic *Arabidopsis* and bermudagrass hairy roots, while knocking down *CdWRKY2* expression via virus‐induced gene silencing increased cold susceptibility. RNA sequencing showed that overexpression of *CdWRKY2* in *Arabidopsis* activated the expression of genes involved in sucrose synthesis and metabolism, including *sucrose synthase 1* (*AtSUS1*) and *sucrose phosphate synthase 2F* (*AtSPS2F*). *CdSPS1*, the homology gene of *AtSPS2F* in bermudagrass, was subsequently proven to be the direct target of CdWRKY2 by yeast one‐hybrid, electrophoretic mobility shift assay, and transient expression analysis. As expected, overexpression of *CdSPS1* conferred cold tolerance in transgenic *Arabidopsis* plants, whereas silencing *CdSPS1* expression enhanced cold sensitivity in bermudagrass. Besides, *CdCBF1* whose expression was dramatically up‐regulated in *CdWRKY2*‐overexpressing bermudagrass hairy roots but down‐regulated in *CdWRKY2*‐silencing bermudagrass both under normal and cold stress conditions was confirmed as another target of CdWRKY2. Collectively, this study reveals that CdWRKY2 is a positive regulator in cold stress by targeting *CdSPS1* and *CdCBF1* promoters and activating their expression to coordinately mediate sucrose biosynthesis and CBF‐signalling pathway, which provides valuable information for breeding cold‐resistant bermudagrass through gene manipulation.

## Introduction

Bermudagrass (*Cynodon dactylon*) is one of the most widely used turfgrass species throughout the world for lawns, sports fields, parks, and slope protection (Fan *et al*., 2014). However, as a representative perennial warm‐season grass, bermudagrass is challenged by low temperature including chilling (0–15 °C) and freezing (<0 °C). Cold stress has been a key environmental factor that adversely influences its growth, development, turf quality, green period, chlorophyll content, and distribution (Fan *et al*., 2014; Liu *et al*., 2016). Therefore, improving cold tolerance is recognised as an important and long‐term target for bermudagrass breeding. To this end, identifying potential genes and uncovering the underlying regulatory networks involved in cold response are imperative.

Cold stress causes several damages at the cellular level, including membrane injury, generation of reactive oxygen species (ROS), and protein denaturation (Ruelland *et al*., 2009). Among them, the plasma membrane injury, which is indicated by electrolyte leakage (EL) and malondialdehyde (MDA) content is thought to be primary adverse effect imposed by cold stress because of its central role in the regulation of various cellular processes (Lyons, 1973; Premachandra *et al*., 1992). As sessile organisms, plants have established sophisticated regulatory mechanisms to cope with cold stress. A myriad of transcription factors (TFs) has been identified to be involved in cold signalling networks, among which C‐repeat binding factors/dehydration responsive element protein 1 (CBFs/DREB1) plays a central role in cold resistance by directly activating the expression of cold responsive (*COR*) genes (Chinnusamy *et al*., 2007). In *Arabidopsis*, three *CBFs*, including *CBF1*, *CBF2*, and *CBF3* have been demonstrated to be essential for cold acclimation (CA) by which plants acquire freezing tolerance upon exposure to advanced low non‐freezing temperatures (Gilmour *et al*., 1998; Jia *et al*., 2016; Park *et al*., 2015; Zhao *et al*., 2016). The *CBFs*, along with their transcriptional activator, inducers of CBF expression 1 (ICE1), constitute ICE1‐CBF‐COR transcriptional cascade which is the most well‐known cold signalling pathway (Chinnusamy *et al*., 2007). In addition to ICE1, a series of TFs including MYB15, CAMTA3, PIF3, EIN3, BZR1 have been identified as upstream regulators of *CBFs* (Agarwal *et al*., 2006; Doherty *et al*., 2009; Jiang et al., 2017a; Kim *et al*., 2013; Li *et al*., 2017; Shi *et al*., 2012). However, investigations on regulatory networks of the cold stress response are mainly focussed on model plants. For bermudagrass, most studies were about the physiology and biochemical measurement of cold stress response rather than decipherment of molecular mechanism (Fan *et al*., 2014; Hu *et al*., 2016; Liu *et al*., 2016; Shi *et al*., 2014). In the previous studies, whole‐genome differentially expressed mRNAs and miRNAs during cold stress response have been identified in bermudagrass (Chen *et al*., 2015; Hu *et al*., 2018); however, due to the lack of an efficient transformation system, there is little progress on functional analysis of cold‐stress responsive genes. Recently, we demonstrated that an ethylene responsive factor CdERF1 from bermudagrass positively regulates cold tolerance through ectopic overexpression of *CdERF1* in *Arabidopsis* plants (Hu *et al*., 2020). Therefore, how to fill the gap in molecular mechanisms of cold stress response in bermudagrass is particularly important.

WRKYs are plant‐specific TFs that have been demonstrated to play a crucial role in growth and development processes such as seed germination (Jiang and Yu, 2009a), flowering (Ma *et al*., 2020; Zhang et al., 2018a), anthocyanin biosynthesis (An *et al*., 2019), and leaf senescence (Jiang *et al*., 2014; Niu *et al*., 2020). Recent studies have also revealed pivotal roles of WRKY TFs in various biotic and abiotic stresses including cold stress (Jiang et al., 2017b; Kim *et al*., 2016; Rushton *et al*., 2010; Sun *et al*., 2019; Yokotani *et al*., 2013; Zhang *et al*., 2019; Zou *et al*., 2010). Most WRKYs have been reported to play a positive role in cold tolerance. For example, the rice *WRKYs*, including *OsWRKY71* and *OsWRKY76* enhance cold resistance (Kim *et al*., 2016; Yokotani *et al*., 2013). Consistently, overexpression of *VaWRKY12* and *VaWRKY33* confer cold resistance in transgenic *Arabidopsis* and grapevine callus (Sun *et al*., 2019; Zhang *et al*., 2019). Niu *et al*. (2012) reported that transgenic *Arabidopsis* plants overexpressing *TaWRKY19* results in improved tolerance to freezing stress as well. On the contrary, *AtWRKY34* which might be involved in the *CBF* signal cascade negatively modulates the cold response of mature pollen (Zou *et al*., 2010). In bermudagrass, a total of 23 WRKY TFs displayed cold‐induced expression patterns (Chen *et al*., 2015). However, the functions of *CdWRKYs* in cold stress response remain largely unknown.

Soluble sugars are considered to play protective roles in cold stress, given that they not only maintain osmotic pressure but also function as signalling molecules regulating the expression of cold‐responsive genes during cold stress (Klotke *et al*., 2004; Rekarte‐Cowie *et al*., 2008). As an important soluble sugar, sucrose accumulates noticeably under cold stress, which is accompanied by the increased transcription of genes encoding sucrose biosynthesis enzymes, such as sucrose synthase (SS), sucrose phosphate synthase (SPS), and invertase (Ruan, 2014). The sucrose content is reported to have a positive relationship with cold tolerance (Jitsuyama *et al*., 2002), which is further demonstrated by the finding that overexpression of *SPS* improved freezing tolerance after CA in transgenic *Arabidopsis* (Strand *et al*., 2003). However, the upstream regulatory TFs important for *SPS* activation under cold stress remain to be identified either in the model or non‐model plants.

*CdWRKY2* whose expression was significantly induced by both chilling and freezing with CA was identified according to our previous transcriptome data (Chen *et al*., 2015). To further investigate the role of *CdWRKY2* in cold stress, function analysis of *CdWRKY2* was performed in this study. We found that CdWRKY2 positively regulates cold tolerance through coordinately activating *CdSPS1*‐involved sucrose synthesis and *CdCBF1*‐dependent signalling pathways. Our results unravel the mechanism of CdWRKY2‐mediated cold resistance, and provide new genetic resources for enhancing cold tolerance in bermudagrass breeding.

## Results

### Identification and characterisation of cold‐responsive *CdWRKY2* in bermudagrass

**Figure 1:**
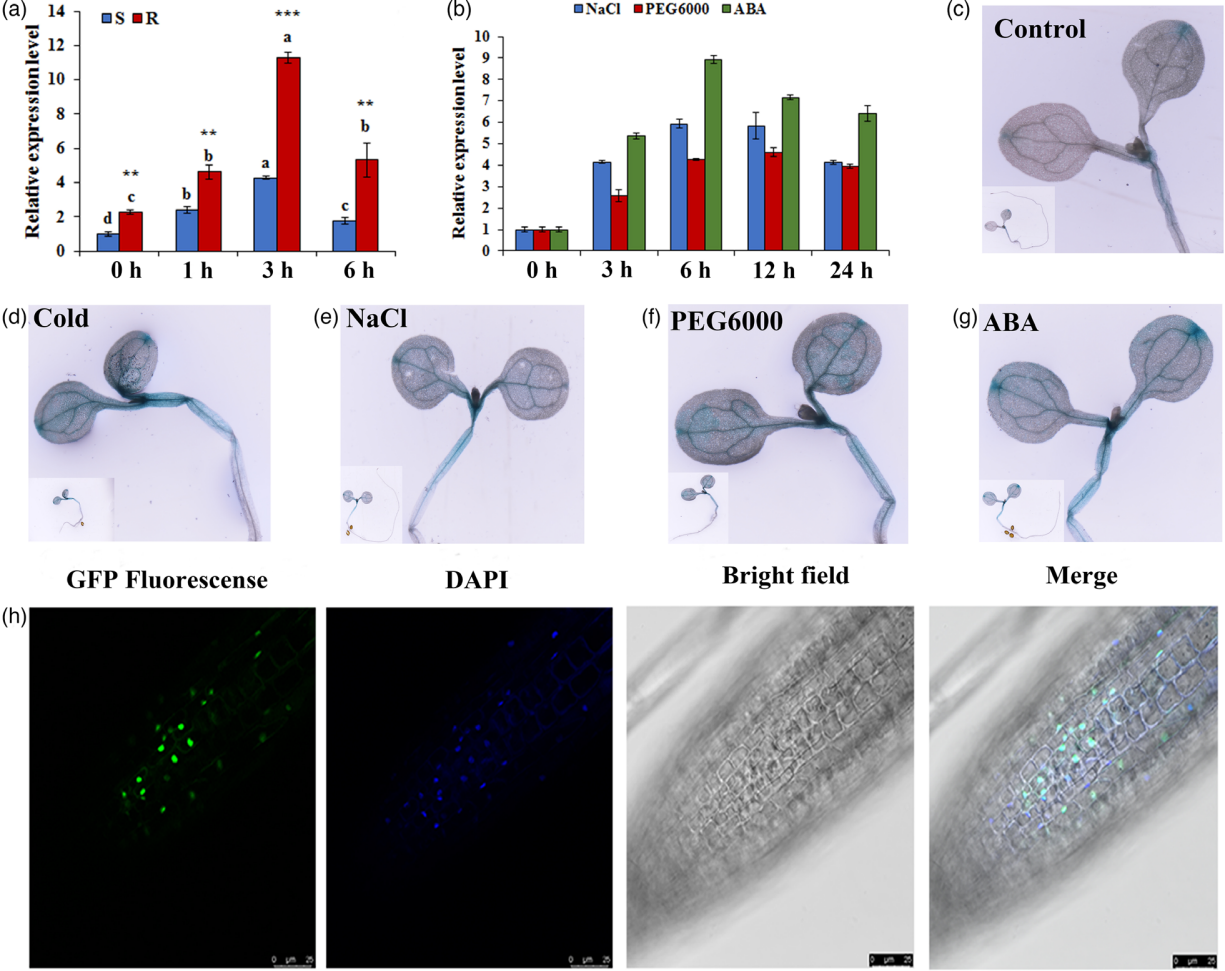
Expression patterns and subcellular localisation of CdWRKY2. (a) Time‐course changes in expression levels of *CdWRKY2* in response to 4 °C cold stress in cold‐resistant (R) and cold‐sensitive (S) bermudagrass. (b) Time‐course changes in expression levels of *CdWRKY2* in response to 200 mM NaCl, 25% PEG6000, and 100 μM ABA in cold‐resistant bermudagrass. (c–g) GUS staining of *ProCdWRKY2:GUS* transgenic *Arabidopsis* seedlings treated with control (c), 4 °C (d), 200 mM NaCl (e), 25% PEG6000 (f), and 100 μM ABA (g). (h) Subcellular localisation of CdWRKY2 in transgenic *Arabidopsis* plants. *CdACTIN2* was used as normalisation controls for quantitative real time‐PCR (qRT‐PCR). The error bars indicate the standard deviation (SD) values while different letters indicate significant statistical differences at among the treatments in two phenotypes according to Duncan’s multiple range tests, respectively (*n* = 3, *P* < 0.05). Asterisks indicate significant differences between the R and S genotypes under the same treatment time according to Student’s *t*‐test (*n* = 3, **P < 0.01, *** *P* < 0.001).

According to our previous transcriptome analysis (Chen *et al*., 2015), a *CdWRKY* (Comp160681_c0) whose expression was significantly induced after both chilling and freezing treatments was screened. Subsequently, a 1683‐bp coding sequence (CDS) was isolated from cold‐resistant bermudagrass by rapid amplification of cDNA ends (RACE), encoding 560 amino acids with a highly conserved WRKY domain followed by a C_2_H_2_‐zinc‐finger motif in the N‐terminus, which belonged to group II (Figure S1a). Phylogenetic analysis suggested that Comp160681_c0 exhibited the highest homology (82.89%) with Sorghum bicolour WRKY2 (SbWRKY2) (Figure S1b). Hereafter, Comp160681_c0 was named *C*. *dactylon WRKY2* (*CdWRKY2,* Genbank accession number: OL472363). To confirm whether *CdWRKY2* is truly a cold‐responsive gene, the expression pattern of *CdWRKY2* was further analysed by quantitative real time‐PCR (qRT‐PCR). Consistent with the RNA sequencing (RNA‐seq) data, the transcript levels of *CdWRKY2* were dramatically up‐regulated in both genotypes after exposure to 1, 3, and 6 h of low temperature, especially in the cold‐resistant genotype. Notably, the *CdWRKY2* expression in cold‐resistant bermudagrass was 2.7‐fold higher than that in the cold‐sensitive one after 3 h of cold treatment (Figure 1a). To further reveal these differentially expressed profiles, the promoter regions, a total of 902‐bp and 904‐bp in length, were cloned from cold‐resistant and ‐sensitive genotypes, respectively. A total of 5 SNP variations were detected between two promoter regions (Figure S2), which may contribute to altered expression levels of *CdWRKY2* between the two genotypes. Apart from cold stress, the transcript levels of *CdWRKY2* were increased by abscisic acid (ABA) and other abiotic stresses, including salt and dehydration in the cold‐resistant bermudagrass as well (Figure 1b). To further verify the expression pattern of *CdWRKY2*, we obtained transgenic *Arabidopsis* plants containing a *GUS* reporter gene driven by the *CdWRKY2* promoter which was cloned from a cold‐resistant genotype. The GUS staining analysis showed that GUS activity was activated by cold stress, as well as NaCl, PEG6000, and ABA treatments (Figure 1c–g), which was consistent with the above qRT‐PCR results (Figure 1a,b).

Subsequently, to determine the subcellular localisation of CdWRKY2 protein, the *35S:CdWRKY2*‐eGFP fusion expression vector was transformed into *Arabidopsis*, and the GFP signal was examined using confocal microscopy. The GFP signal was located in the nucleus, which was further validated by DAPI staining, indicating that the CdWRKY2 is exclusively located in the nucleus (Figure 1h).

### Overexpression of *CdWRKY2* enhances cold tolerance in transgenic *Arabidopsis*

**Figure 2:**
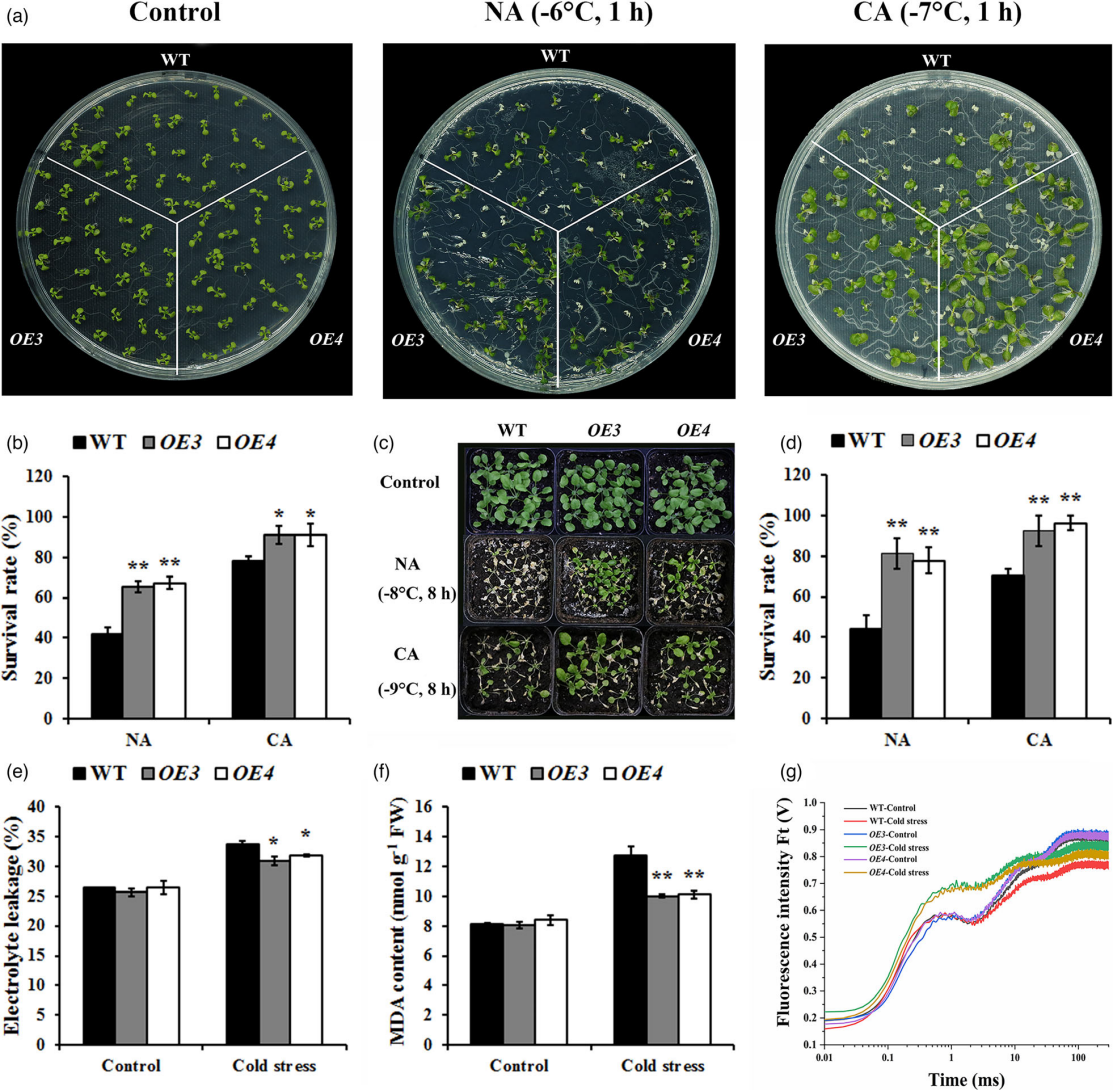
Overexpression of *CdWRKY2* confers cold tolerance in transgenic *Arabidopsis*. (a,b) Freezing phenotypes (a) and survival rates (b) of 10‐day‐old *CdWRKY2*‐overexpressing and WT *Arabidopsis* seedlings on petri dishes with or without cold acclimation (CA, 7 days at 4 °C). Ten‐day‐old seedlings were subjected to 1 h of freezing treatment at −6 °C for non‐acclimated (NA) and −7 °C for CA, seedlings were transferred to 4 °C for 24 h, then recovered at 22 °C for an additional 3 days. (c,d) Freezing phenotypes (c) and survival rates (d) of *CdWRKY2*‐overexpressing *Arabidopsis* and WT plants in soil with or without CA. Three‐week‐old plants were exposed to 8 h of freezing treatment at −8 °C for NA and −9 °C for CA followed by recovery at 22 °C for 3 days. (e–g) Electrolyte leakage (EL) (e), malondialdehyde (MDA) content (f), and the chlorophyll a fluorescence transient (OJIP) curves (g) of WT and *CdWRKY2*‐overexpressing *Arabidopsis* under normal (22 °C for 7 days) and cold stress conditions (4 °C for 7 days). Asterisks indicate significant differences between the transgenic lines and WT under the same growth conditions according to Student’s *t*‐test (*n* ≥ 3, *P < 0.05, **P < 0.01).

To understand the biological function of *CdWRKY2* in regulating cold tolerance, two *CdWRKY2‐*overexpressing *Arabidopsis* lines (*OE3* and *OE4*) with relative high expression were selected for assessing cold tolerance (Figure S3a). No conspicuous difference in phenotype was observed between transgenic *Arabidopsis* plants and WT under normal conditions. However, 10‐day‐old *CdWRKY2‐*overexpressing *Arabidopsis* improved cold tolerance after freezing treatments regardless of CA (Figure 2a). With CA, the average survival rates of *OE3* and *OE4* increased by 16% and 17% compared with WT, whereas the average survival rates of transgenic plants overexpressing *CdWRKY2* increased by 39% and 36% without CA (Figure 2b). Analogously, the phenotypes and average survival rates of 3‐week‐old transgenic *Arabidopsis* also indicated that overexpression of *CdWRKY2* significantly enhances cold tolerance in transgenic plants (Figure 2c,d).

Subsequently, physiological responses to cold stress were compared between WT and *CdWRKY2‐*overexpressing *Arabidopsis* plants. After 7 days of 4 °C treatment, EL and MDA content which are two representative indicators of cell membrane stability in the transgenic lines were significantly lower than those in WT (Figure 2e,f). Chlorophyll fluorescence intensity can reflect the performance of photosystem II (PSII) in plants’ response to cold stress. Before 4 °C treatment, the chlorophyll a fluorescence transient (OJIP) curves were indistinguishable between transgenic lines and WT. However, cold stress negatively altered OJIP curves in all plants, especially in WT (Figure 2g). Meanwhile, the decreases of F_v_/F_m_ ratios, PI_ABS,_ and PI_total_ values which are important photosynthetic parameters were all rescued by overexpressing *CdWRKY2* under cold stress (Figure S3b–d). Taken together, these results indicated that ectopic expression of *CdWRKY2* enhances cold tolerance in transgenic *Arabidopsis* plants.

### Silencing of *CdWRKY2* expression in bermudagrass increases hypersensitivity to cold stress

**Figure 3:**
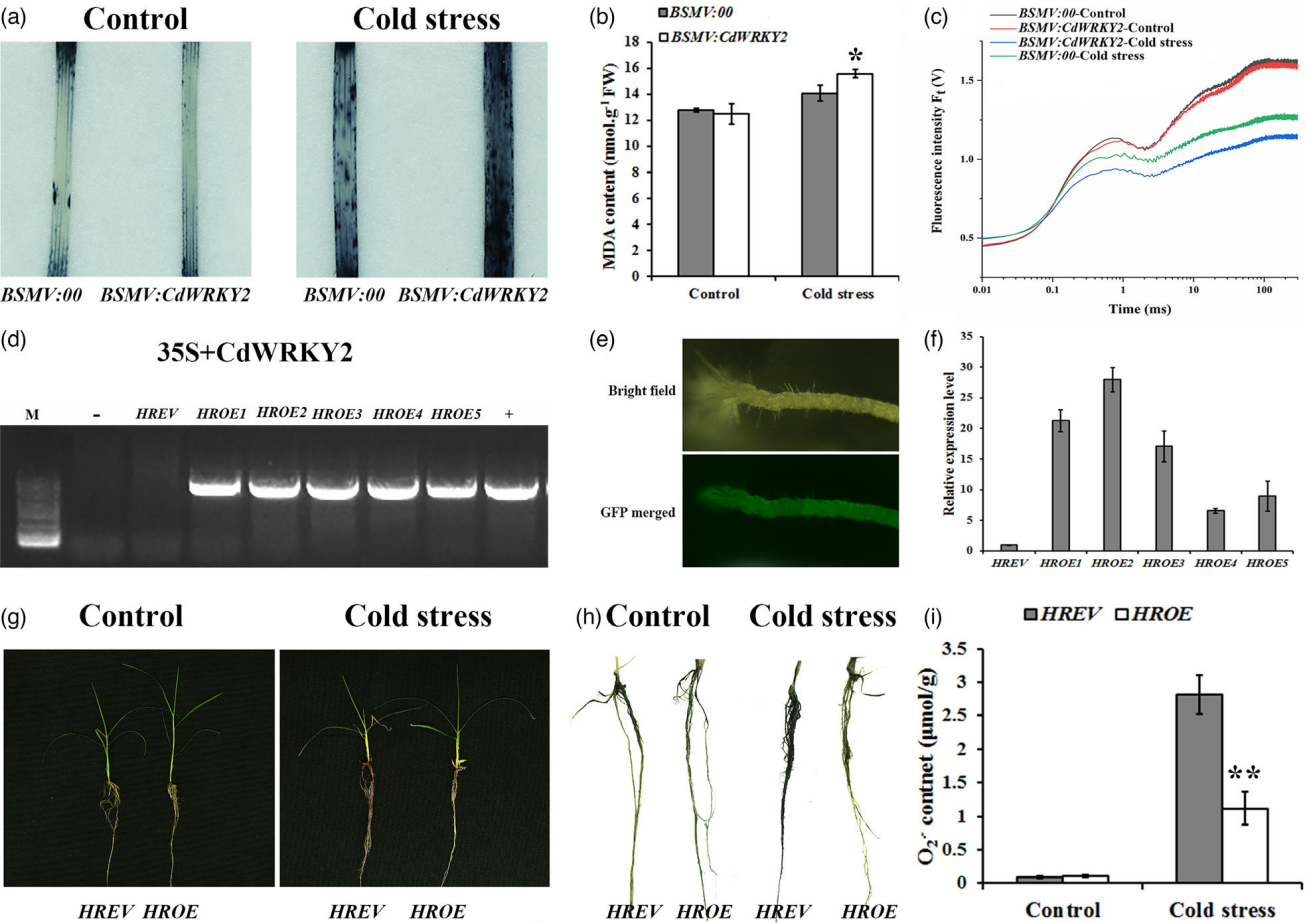
CdWRKY2 positively regulates cold tolerance in bermudagrass. (a–c) Histochemical staining with nitro blue tetrazolium (NBT) (a), MDA concentrations (b), and OJIP curves (c) of *BSMV:00* and *BSMV:CdWRKY2* bermudagrass under normal and cold stress conditions (4 °C for 7 days). *BSMV:CdWRKY2*, *CdWRKY2*‐silencing bermudagrass generated by virus‐induced gene silencing (VIGS); *BSMV:00*, control bermudagrass generated by VIGS. (d) PCR identification of *35S:CdWRKY2* in transgenic bermudagrass hairy roots using 35S‐F/CdWRKY2‐eGFP‐R primers. +, 35S:CdWRKY2‐eGFP (used as a positive control), −, ddH2O; *HREV*: bermudagrass hairy root containing empty vector (pBI121‐eGFP*)*; *HROE*: bermudagrass hairy root overexpressing *CdWRKY2*. (e) GFP signals in *CdWRKY2‐*overexpressing hairy roots of bermudagrass. (f) Relative expression levels of *CdWRKY2* in *HREV* and *HROEs*. (g) Phenotypes of single seedling of *HREVs* and *HROEs* under normal and cold stress conditions (4 °C for 7 days). (h,i) Histochemical staining with NBT (h) and O_2_^−^ content (i) in hairy roots of *HREV* and *HROE* under normal and cold stress conditions (4 °C for 7 days). The error bars indicate the SD values from at least three repetitions of each treatment. Asterisks indicate significant differences between *BSMV:00* and *BSMV:CdWRKY2* under the same growth conditions (*n* ≥ 3, *P < 0.05 or **P < 0.01).

To elucidate the function of *CdWRKY2* in regulating cold tolerance in bermudagrass, the virus‐induced gene silencing (VIGS) system was employed. As shown in Figure S4a, the transcription level of *CdWRKY2* was substantially decreased by about 50% in *BSMV:CdWRKY2* compared with that of control plants (*BSMV:00*), which were only infiltrated with empty vectors. Under normal conditions, the *BSMV:CdWRKY2* and *BSMV:00* bermudagrass plants were morphologically indistinguishable. However, upon exposure to 21 days of 4 °C cold stress treatment, leaves of *BSMV:CdWRKY2* displayed more severely wilting and necrosis in comparison with those of *BSMV:00* (Figure S4b), which was supported by the results of histochemical staining with nitro blue tetrazolium (NBT; Figure 3a). Consistently, MDA content increased after cold treatment, to a greater degree in *BSMV:CdWRKY2* plants (Figure 3b). Besides, PSII efficiency which was indicated by OJIP curves, F_v_/F_m_ ratios, PI_ABS,_ and PI_total_ values decreased significantly in *BSMV:CdWRKY2* compared with that in control after cold treatment, although it was indistinguishable between *BSMV:CdWRKY2* and *BSMV:00* under normal conditions (Figure 3c and Figure S4c–e). Subsequently, expression levels of cold marker genes, such as *CdABF1*, *CdCBF1*, *CdLEA3*, and *CdCOR440* were further analysed. As a result, *CdABF1*, *CdCBF1*, *CdLEA3*, and *CdCOR440* were all down‐regulated by knocking down the expression of *CdWRKY2* with or without cold stress treatment in bermudagrass (Figure S4f–i). Taken together, these results indicate that the knockdown of *CdWRKY2* expression enhances cold susceptibility in bermudagrass.

### *Agrobacterium rhizogenes*‐mediated overexpression of *CdWRKY2* confers cold tolerance in bermudagrass with transgenic hairy roots

*Agrobacterium* *tumefaciens*‐mediated genetic transformation in bermudagrass is still immature, which restricts gene functional analysis in vivo. Here, *A*. *rhizogenes*‐mediated transformation was successfully developed in bermudagrass. The transgenic hairy roots were identified by genomic PCR, GFP signal detection, and qRT‐PCR (Figure 3d–f). Transgenic hairy roots with relatively high expression of *CdWRKY2* were selected for function characterisation, while transgenic hairy roots transferring an empty vector were used as a control (Figure 3f). No phenotypic differences were observed between the plants with *CdWRKY2‐*overexpressing hairy roots and control plants under normal conditions. After 10 days of cold stress (4 °C day/night temperature) treatment, the leaves of control plants displayed slightly wilting and necrosis, while no obvious damages were observed in plants with *CdWRKY2‐*overexpressing hairy roots (Figure 3g). NBT staining result indicated that control hairy roots displayed more severe damages in comparison with transgenic hairy roots (Figure 3h), which was consistent with the result of O_2_^−^ content (Figure 3i). Consistently, cold marker genes, such as *CdABF1*, *CdCBF1*, *CdLEA3*, and *CdCOR440*, exhibited significantly higher expression levels in three transgenic lines regardless of cold stress treatment (Figure S5a–d). These results indicated that CdWRKY2 is indeed a positive regulator in bermudagrass against cold stress.

### CdWRKY2 activates the expression of sucrose‐related genes

**Figure 4:**
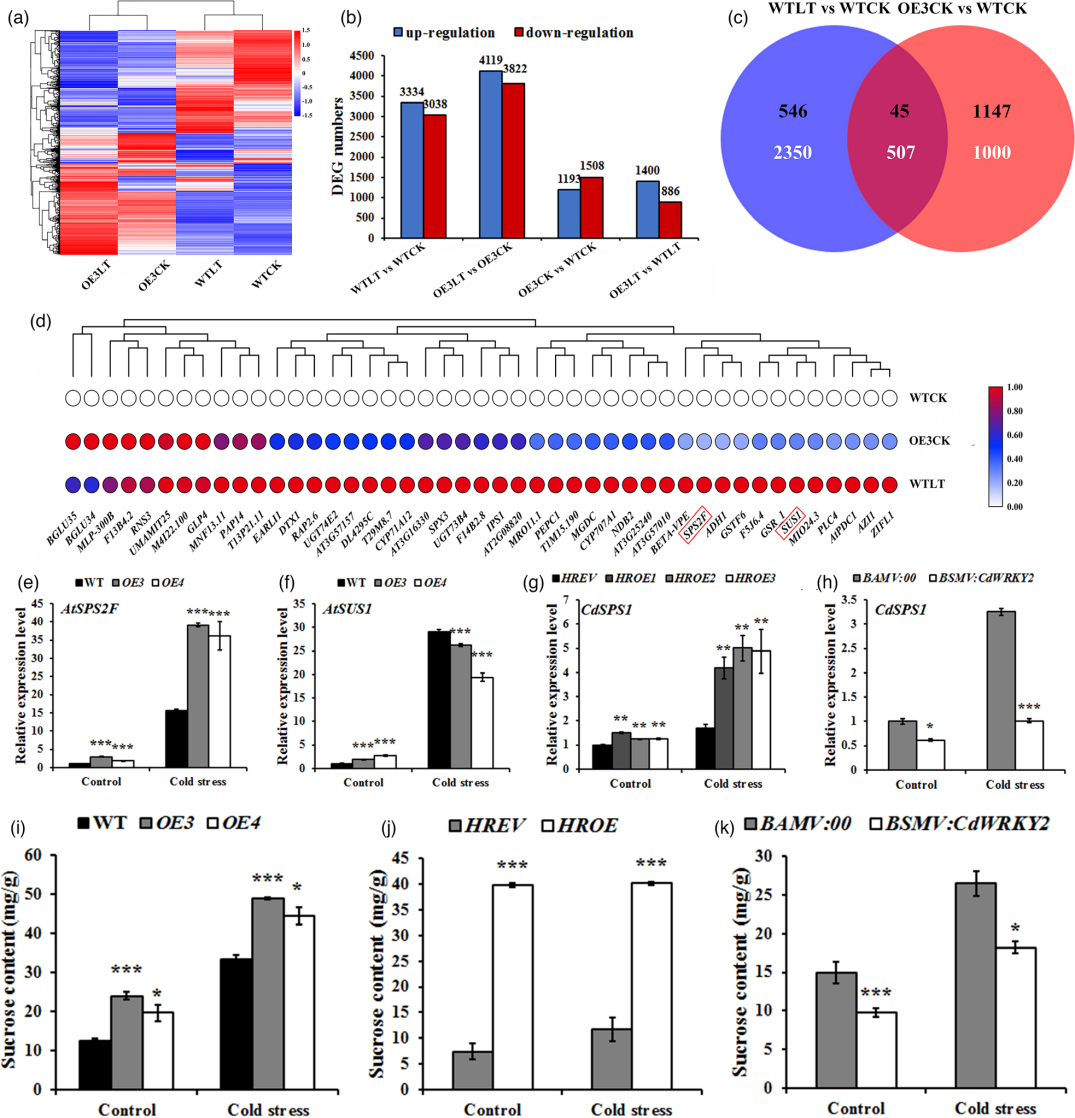
Expression levels of sucrose synthesis genes and concentrations of sucrose changed dependent on the presence of CdWRKY2. (a) Heap map of differentially expressed genes (DEGs) in WT and *CdWRKY2‐*overexpressing *Arabidopsis* after 6 h of 4 °C treatment. (b) The number of DEGs in four comparisons including WTLT vs WTCK, OE3LT vs OE3CK, OE3CK vs WTCK, and OE3LT vs WTLT. WTLT, 7‐day‐old seedlings of WT after 6 h of 4 °C cold stress; WTCK, 7‐day‐old seedlings of WT before 6 h of 4 °C cold stress; OE3LT, 7‐day‐old seedlings of *CdWRKY2*‐overexpressing transgenic *Arabidopsis* after 6 h of 4 °C cold stress; OE3CK, 7‐day‐old seedlings of *CdWRKY2‐*overexpressing transgenic *Arabidopsis* before 6 h of 4 °C cold stress. (c) The Venn diagram of DEGs in comparisons WTLT vs WTCK and OE3CK vs WTCK. The black and white numbers represent up‐regulated and down‐regulated genes, respectively. (d) Hierarchical clustering analysis of 45 up‐regulated DEGs in WTCK, OE3CK, and WTLT. Red, blue and white elements in the matrix indicate up‐regulated, no change, and down‐regulated genes, respectively. (e‐f) Expression levels of sucrose synthesis genes *AtSPS2F* (e) and *AtSUS1* (f) in WT and *CdWRKY2‐*overexpressing *Arabidopsis* plants after 6 h of 4 °C cold treatment. (g,h) Expression levels of *CdSPS1* in *CdWRKY2‐*overexpressing bermudagrass hairy roots (g) and *CdWRKY2‐*silencing bermudagrass plants (h). (i–k) Sucrose contents in *CdWRKY2‐*overexpressing *Arabidopsis* (i), *CdWRKY2‐*overexpressing hairy roots of bermudagrass (j), and *CdWRKY2‐*silencing bermudagrass (k) under control and cold stress (4 °C for 7 days) conditions. The error bars indicate the SD values from at least three repeats of each treatment. Asterisks indicate significant differences (*n* ≥ 3, *P < 0.05, **P < 0.01, ***P < 0.001) between the transgenic lines and control plants under the same growth conditions according to Student’s *t*‐test.

To reveal the underlying regulatory mechanism of CdWRKY2 in plant cold response, RNA‐Seq analysis was performed using 7‐day‐old seedlings of WT and *CdWRKY2‐*overexpressing transgenic (*OE3*) *Arabidopsis* before or after 6 h of 4 °C treatments, named WTCK (7‐day‐old seedlings of WT before 6 h of 4 °C cold stress), OE3CK (7‐day‐old seedlings of *CdWRKY2‐*overexpressing transgenic *Arabidopsis* before 6 h of 4 °C cold stress), WTLT (7‐day‐old seedlings of WT after 6 h of 4 °C cold stress), and OE3LT (7‐day‐old seedlings of *CdWRKY2‐*overexpressing transgenic *Arabidopsis* after 6 h of 4 °C cold stress), respectively. The hierarchical clustering analysis showed that cold stress dramatically altered gene expression profiles both in WT and transgenic plants (Figure 4a). A total of 6372 (including 3334 up‐regulated and 3038 down‐regulated), 7941 (including 4119 up‐regulated and 3822 down‐regulated), 2701 (including 1193 up‐regulated and 1508 down‐regulated), and 2286 (including 1400 up‐regulated and 886 down‐regulated) differentially expressed genes (DEGs) were identified in comparison WTLT vs WTCK, OE3LT vs OE3CK, OE3CK vs WTCK, and OE3LT vs WTLT, respectively (Figure 4b). The Kyoto Encyclopaedia of Genes and Genomes (KEGG) pathway analysis showed that these DEGs were significantly enriched in starch and sucrose metabolism, photosynthesis‐antenna proteins, carbon metabolism, and glyoxylate and dicarboxylate metabolism (Figure S6). Gene ontology (GO) analysis displayed that these DEGs were highly involved in the biological processes including response to oxidative stress, response to cold, and cell wall organisation (Figure S7). To further narrow down the scope of candidate genes involved in CdWRKY2‐mediated cold stress, a Venn diagram analysis was performed using DEGs with a fourfold difference in comparisons OE3CK vs WTCK and WTLT vs WTCK. A total of 45 up‐regulated and 507 down‐regulated DEGs were overlapped between OE3CK vs WTCK and WTLT vs WTCK (Figure 4c). Among the 45 up‐regulated DEGs, there were several genes responsive to oxidative stress, including two *UDP‐glycosyltransferases* (*UGT74E2* and *UGT73B4*), one *Glutathione transferase* (*GSTF6*), one *Germin‐like protein* (*GLP4*), and two *beta‐glucosidases* (*BGLU34* and *BGLU35*). Most importantly, two up‐regulated genes, *AtSPS2F* and *AtSUS1* (Figure 4d), involved in sucrose synthesis and metabolism process that has been reported to be associated with cold tolerance were identified (Zhao *et al*., 2020). The expression patterns of *AtSPS2F* and *AtSUS1* were subsequently validated by qRT‐PCR (Figure 4e,f). The *AtSPS2F* expression was significantly up‐regulated in *CdWRKY2‐*overexpressing *Arabidopsis* plants under normal and cold stress conditions (Figure 4e). However, unlike with *AtSPS2F*, the *AtSUS1* expression was significantly lower in *CdWRKY2‐*overexpressing *Arabidopsis* plants under cold stress compared with that in WT (Figure 4f). We thus further focussed on whether the expression of *CdSPS1,* which is the homology gene of *AtSPS2F* in bermudagrass is affected by CdWRKY2. The expression of *CdSPS1* was performed in *CdWRKY2‐*silencing bermudagrass and *CdWRKY2‐*overexpressing bermudagrass hairy roots under normal and low‐temperature conditions. As expected, mRNA abundance of *CdSPS1* was significantly increased in the bermudagrass plants with *CdWRKY2‐*overexpressing hairy roots relative to control roots (Figure 4g). By contrast, transcript levels of *CdSPS1* were dramatically down‐regulated in *BSMV:CdWRKY2* as compared with *BSMV:00* with or without cold stress (Figure 4h). Collectively, sucrose synthesis genes including *AtSPS2F* and *CdSPS1* might be induced by CdWRKY2 directly or indirectly during cold stress.

### CdWRKY2 positively regulates sucrose accumulation

Given that the expression levels of genes involved in sucrose biosynthesis were regulated by CdWRKY2, sucrose contents were further measured in *CdWRKY2‐*overexpressing, *CdWRKY2‐*silencing, and control plants. Consequently, sucrose concentrations were induced after cold treatment, and sucrose contents in *Arabidopsis* lines *OE3* and *OE4* were significantly higher than those of WT with or without cold stress treatment (Figure 4i). Consistently, sucrose contents in *CdWRKY2* transgenic hairy roots were fivefold and 3.5‐fold higher than those of control roots under control and cold stress conditions, respectively (Figure 4j). In contrast, the *BSMV:CdWRKY2* bermudagrass exhibited significantly lower sucrose concentrations relative to *BSMV:00* plants (Figure 4k). Taken together, our results indicated that CdWRKY2 positively regulates cold stress likely through inducing sucrose accumulation.

### CdWRKY2 activates *CdSPS1* expression by directly binding to the *CdSPS1* promoter

**Figure 5:**
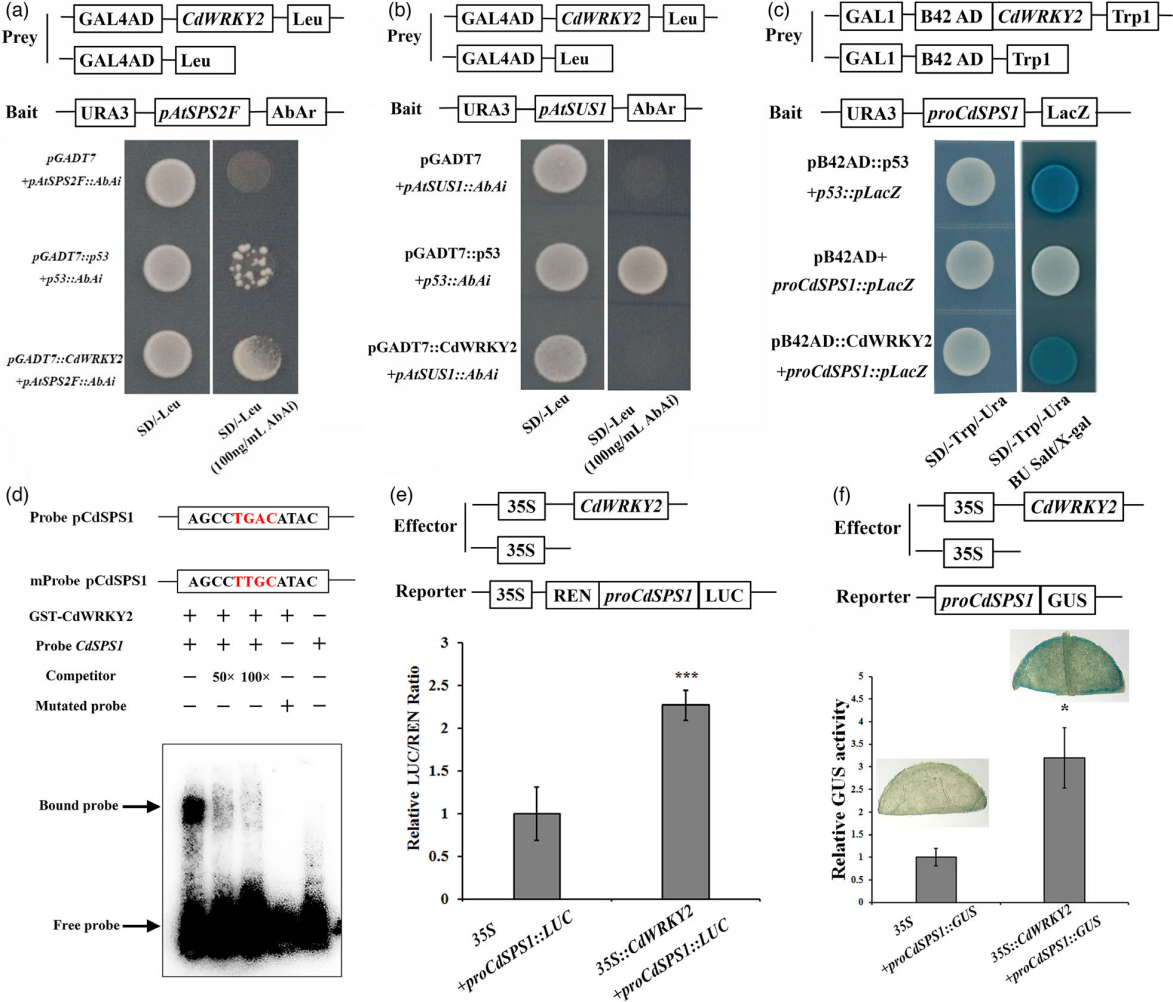
CdWRKY2 directly binds to the promoters of *AtSPS2F* and *CdSPS1* and activates their expression levels. (a,b) Yeast one‐hybrid analysis, using pGADT7‐CdWRKY2 as the prey, *pAtSUS1‐AbAi* (a) and *pAtSPS2F‐AbAi* (b) as the baits, pGADT7*‐*p53 and *p53‐AbAi* as the positive controls. (c) Yeast one‐hybrid analysis, using pB42AD‐CdWRKY2 as the prey, *ProCdSPS1‐pLacZ* as the baits, pB42AD‐p53 and *p53‐pLacZ* as the positive controls. (d) Electrophoretic mobility shift assay (EMSA) of the interaction between fusion protein GST‐CdWRKY2 and the *CdSPS1* promoter. The purified GST‐CdWRKY2 protein was incubated with the biotin‐labelled probes containing WT or mutated W‐box element. The unlabelled WT probe (50× and 100×) was used as a competitor. The bound DNA‐protein complex is indicated by the arrows. +, presence; −, absence. (e) Luciferase activity analysis using *35S:CdWRKY2* as the effector and *ProCdSPS1:LUC* as a reporter. The REN and LUC are Renilla luciferase and firefly luciferase, respectively. LUC:REN ratio of the control (tobacco leaves co‐transformed with the reporter and the empty effector vector) was taken as 1 for normalisation. (f) Transient β‐glucuronidase expression analysis, using *35S:CdWRKY2* as the effector and *ProCdSPS1:GUS* as reporter. GUS activity of the control (tobacco leaves co‐transformed with the reporter and the empty effector vector) was taken as one for normalisations. The error bars indicate the SD values from at least three repetitions of each treatment. Asterisks (*n* = 5, *P < 0.05, ***P < 0.001) indicate significant differences compared with the control, respectively (Student’s *t*‐test).

To explore whether CdWRKY2 activates the expression of *AtSPS2F* and *AtSUS1* directly in *Arabidopsis*, a yeast one‐hybrid assay (Y1H) was carried out. As a result, yeast strain Y1H Gold harbouring *pAtSPS2F*‐AbAi and pGADT7‐CdWRKY2 vectors could grow on SD/‐Leu medium supplemented with 100 ng/mL aureobasidin A (AbA), which could suppress the basal expression in the Y1H Gold harbouring *pAtSPS2F*‐AbAi, suggesting the interaction between CdWRKY2 and *AtSPS2F* promoter (Figure 5a). However, CdWRKY2 failed to activate the *AbAr* reporter gene driven by the *AtSUS1* promoter (Figure 5b). Therefore, the homology gene of *AtSPS2F* in bermudagrass was chosen for further analysis. Because 1000 ng/mL AbA was still unable to suppress the basal expression in the Y1H Gold harbouring *proCdSPS1*‐AbAi, another Y1H system (pB42AD/pLacZi) was used alternatively. The result showed that GAD‐CdWRKY2 fusion protein, instead of GAD (GAL1 transcriptional activation domain, AD), activated the *LacZ* reporter gene driven by the *CdSPS1* promoter which was cloned from cold‐resistant genotype (Figure 5c), indicating that CdWRKY2 can bind to *CdSPS1* promoter directly. As we know, WRKY TFs activate or suppress target gene expression by binding to the W‐box (T/CTGACC/T) or its core motif (TGAC). Expectedly, a TGAC core motif was found in upstream of the *CdSPS1* promoter region (−860~−863 bp).

To verify whether CdWRKY2 binds to the W‐box of the *CdSPS1* promoter, EMSA was conducted in vitro. The formation of the protein‐DNA complex was only observed when the fusion protein was incubated with a labelled probe containing a wild‐type (WT) W‐box element, which was reduced by adding the unlabelled competitor. Besides, no bind shift was detected when the mutated probe was incubated with the fusion protein. (Figure 5d). These results confirmed that CdWRKY2 binds to the W‐box element of the *CdSPS1* promoter.

Subsequently, a dual‐luciferase reporter assay was performed to further verify the binding of CdWRKY2 to the *CdSPS1* promoter. The firefly luciferase (LUC) driven by the *CdSPS1* promoter (*proCdSPS1:LUC*) and renilla luciferase (REN) driven by 35S promoter (*35S:REN*) were transformed transiently into tobacco leaves with *35S:CdWRKY2* or empty vector (pMD35S), respectively. As shown in Figure 5e, the LUC activity, which is indicated by LUC/REN ratio in leaves co‐expressing *35S:CdWRKY2* and *proCdSPS1‐LUC* were about twofold higher than those in the control leaves infiltrated with the pMD35S and *proCdSPS1‐LUC*. Additionally, a transient GUS expression assay was performed as well. The GUS staining was stronger in the tobacco leaf co‐transformed with the *35S:CdWRKY2* and *proCdSPS1:GUS*, which was further supported by the GUS activity assays (Figure 5f). Collectively, all data indicated that CdWRKY2 can directly bind to the *CdSPS1* promoter.

### *CdSPS1* positively regulates cold tolerance in transgenic plants

**Figure 6:**
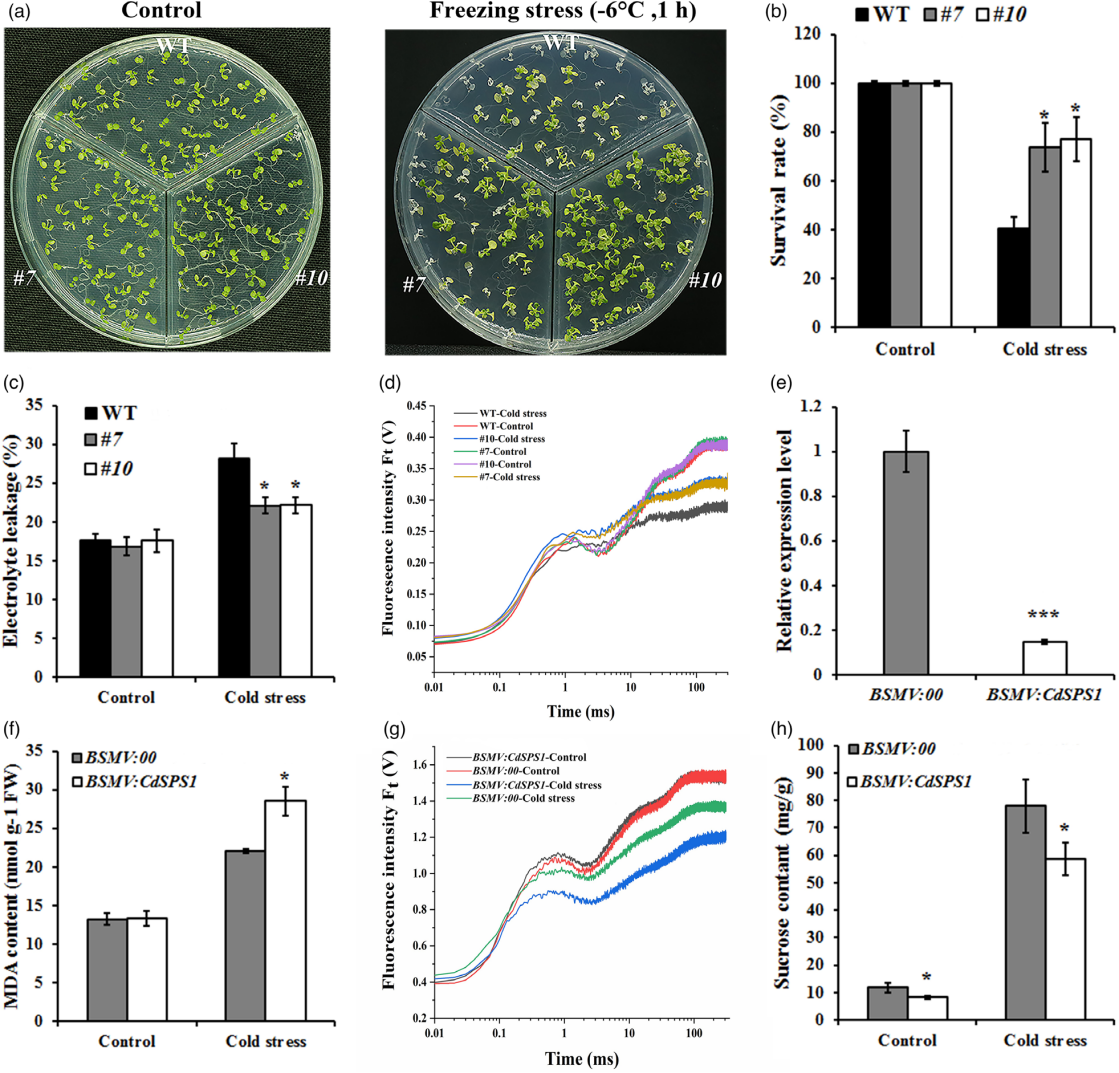
Functional analysis of *CdSPS1* in response to cold stress of *Arabidopsis* and bermudagrass. (a,b) Phenotypes (a) and survival rates (b) of the *CdSPS1‐*overexpressing and WT *Arabidopsis* plants under normal and freezing treatments (−6 °C for 1 h). (c,d) EL (c) and OJIP curves (d) of WT and *CdSPS1‐*overexpressing transgenic *Arabidopsis* after cold treatments (4 °C for 7 days). (e) Relative expression levels of *CdSPS1* in control (*BSMV:00*) and *CdSPS1‐*silencing bermudagrass (*BSMV:CdSPS1*). (f–h) MDA concentrations (f), OJIP curves (g), and sucrose contents (h) of *BSMV:00* and *BSMV:CdSPS1* bermudagrass under normal and cold stress (4 °C for 7 days) conditions. The error bars indicate the SD values from at least three repetitions of each treatment. Asterisks indicate significant differences (*n* ≥ 3, *P < 0.05, ***P < 0.001) between the transgenic lines and control plants under the same growth conditions.

To investigate whether *CdSPS1* plays a role in cold tolerance, two *CdSPS1*‐overexpressing *Arabidopsis* lines (*#7* and *#10*) with relative high expression levels were selected for further analysis (Figure S8a). Ten‐day‐old WT and transgenic *Arabidopsis* plants were subjected to freezing treatments (−6 °C, 1 h) without CA. Expectedly, *CdSPS1*‐overexpressing plants significantly improved cold tolerance of transgenic *Arabidopsis* seedlings (Figure 6a), as indicated by significantly higher survival rates (Figure 6b). The subsequent physiological responses to cold stress further demonstrated the prominent role of *CdSPS1* in cold resistance. After 7 days of 4 °C treatment, the EL in the *CdSPS1*‐overexpressing lines was significantly lower than those in WT, although cold stress led to the increase of EL in all plants (Figure 6c). Meanwhile, the decreased PSII efficiency reflected by the OJIP curve, F_v_/F_m_ ratios, PI_ABS,_ and PI_total_ values were all repaired by overexpressing *CdSPS1* under cold stress condition (Figure 6d and Figure S8b–d).

In addition, the function of *CdSPS1* in regulating cold tolerance in bermudagrass was further validated by knocking down *CdSPS1* expression using the VIGS system (Figure 6e). After 4 °C cold stress treatment, more severely wilting and necrosis leaves were observed in *BSMV:CdSPS1* compared with *BSMV:00* (Figure S9a), which was evidenced by the results of MDA contents, OJIP curves, F_v_/F_m_, PI_ABS,_ and PI_total_ values (Figure 6f,g and Figure S9b–d). Subsequently, the sucrose contents were measured in *BSMV:00* and *BSMV:CdSPS1* plants, showing that sucrose concentrations were indeed decreased by knocking down *CdSPS1* expression (Figure 6h). Taken together, these results indicated that *CdSPS1* plays a positive role in cold tolerance both in *Arabidopsis* and bermudagrass.

### CdWRKY2 activates *CdCBF1* expression by directly binding to *CdCBF1* promoter

**Figure 7:**
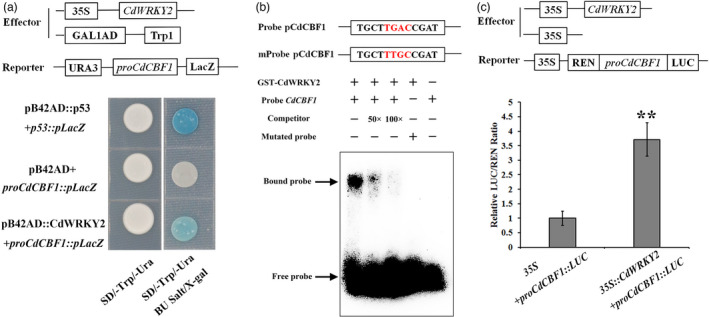
CdWRKY2 directly binds to the *CdCBF1* promoter and activates its expression level. (a) Yeast one‐hybrid analysis, using pB42AD‐CdWRKY2 as the prey, *ProCdCBF1‐pLacZ* as the bait, and pB42AD‐p53 and *p53‐pLacZ* as the positive controls. (b) EMSA analysis of the interaction between fusion protein GST‐CdWRKY2 and the *CdCBF1* promoter. The purified GST‐CdWRKY2 protein was incubated with the biotin‐labelled probes containing WT or mutated W‐box elements. The unlabelled probe (50× and 100×) was used as a competitor. The bound DNA‐protein complex is indicated by the arrows. +, presence; −, absence. (c) Luciferase activity analysis using *35S:CdWRKY2* as the effector and *ProCdCBF1:LUC* as a reporter. The error bars indicate the SD values from at least three repetitions of each treatment. Asterisks (*n* = 5, **P < 0.01) indicate significant differences compared with the control (Student’s *t*‐test).

It is well known that *CBF1* plays a central role in cold resistance, and *CdCBF1* expression was induced in *CdWRKY2*‐overexpressing but suppressed in *CdWRKY2*‐silencing transgenic plants (Figures S4g and S5b), which raised the possibility that *CdCBF1* may be involved in CdWRKY2‐mediated cold stress response. To explore whether CdWRKY2 activates *CdCBF1* expression directly, Y1H, EMSA, and LUC‐based transactivation experiment were carried out. As shown in Figure 7a, the *LacZ* reporter gene driven by the *CdCBF1* promoter was activated by GAD‐CdWRKY2 fusion protein, indicating that CdWRKY2 can bind to *CdCBF1* promoter directly. The subsequent EMSA assay demonstrated that CdWRKY2 specifically binds to the W‐box (−131~−136 bp) in the *CdCBF1* promoter (Figure 7b). Consistently, the LUC activity in leaves co‐expressing *35S:CdWRKY2* and *proCdCBF1‐LUC* was about 3.5‐fold higher than that in the control leaves infiltrated with the pMD35S and *proCdCBF1‐LUC* (Figure 7c). All results indicated that CdWRKY2 can directly bind to the *CdCBF1* promoter and activate its expression.

## Discussion

Cold stress is one of the most common factors that limits the development and geographical distribution of bermudagrass. Increasing evidence has indicated that WRKY TFs play pivotal roles in response to cold stress in many species (Kim *et al*., 2016; Niu *et al*., 2020; Sun *et al*., 2019; Yokotani *et al*., 2013; Zhang *et al*., 2019). However, there is no information about *CdWRKYs* involved in cold stress response as yet. In this study, we identified a cold‐induced *CdWRKY2* based on transcriptome data and qRT‐PCR expression analysis. We found that overexpression of *CdWRKY2* enhanced cold tolerance, while silencing of *CdWRKY2* impaired cold resistance in bermudagrass, indicating that *CdWRKY2* functions as a positive regulator in cold stress response of bermudagrass.

### The *CdWRKY2* expression is induced by cold stress as well as other abiotic stresses

The expression of *CdWRKY2* was significantly induced by cold stress both in cold‐resistant and cold‐sensitive bermudagrass genotypes, and its level was significantly higher in the cold‐resistant genotype than that in the cold‐sensitive one during cold stress (Figure 1a). Interestingly, *CdWRKY2* expression level was 2.6‐fold higher in the cold‐resistant genotype than that in the cold‐sensitive one under normal conditions (Figure 1a), which raises the possibility of variations in *CdWRKY2* promoters between two genotypes. Consequently, a total of 5 SNP variations were detected between two *CdWRKY2* promoters from two genotypes (Figure S2). However, whether the SNP variations result in the differential expression of *CdWRKY2* between two genotypes needs to be further investigated. Apart from cold stress, *CdWRKY2* expression was also induced by salt, drought, and ABA treatments (Figure 1b). Similarly, the expression level of *AtWRKY2* was also increased by NaCl and mannitol treatments (Jiang and Yu, 2009b). As we know, cold, drought, and salinity can lead to water deficit which generates osmotic stress (Zhu, 2016). Moreover, ABA has been suggested to regulate CA by activating osmotic responses (Shi *et al*., 2014). Therefore, we speculate that *CdWRKY2* is likely to play a role in cold‐induced osmotic stress, which may be influenced by ABA signalling.

### CdWRKY2 improves cold tolerance by positively regulating *CdSPS1* expression to accumulate sucrose concentrations

To investigate the regulatory mechanism of *CdWRKY2* during cold stress, transcriptome analysis was conducted between WT and *CdWRKY2‐*overexpressing *Arabidopsis* plants. The data displayed that genes participating in sucrose synthesis and metabolism, especially *AtSPS2F*, were significantly induced in *CdWRKY2‐*overexpressing *Arabidopsis* plants both under normal and cold stress conditions (Figure 4e). Likewise, *CdSPS1* which is the homology gene of *AtSPS2F* was remarkably up‐regulated in *CdWRKY2‐*overexpressing hairy roots but dramatically down‐regulated in *CdWRKY2‐*silencing bermudagrass (Figure 4g–h). Corresponding to the changes in expression levels of *AtSPS2F* and *CdSPS1*, sucrose concentrations were substantially elevated in *CdWRKY2‐*overexpressing transgenic *Arabidopsis* and hairy roots under normal and low‐temperature conditions (Figure 4i,j), while the opposite trend was observed in *CdWRKY2‐*silencing bermudagrass plants (Figure 4k). Recent studies have suggested that sucrose, an osmotic protectant, plays a vital role in abiotic stresses, including cold stress (Zhao *et al*., 2020). Sucrose content prominently increases during CA both in the field and artificial conditions in overwintering evergreens (Liu *et al*., 2020). The increase of sucrose biosynthesis during CA is essential for developing freezing tolerance (Strand *et al*., 2003). Considering SPS mediates the rate‐limiting step of sucrose synthesis (Castleden *et al*., 2004), the SPS‐mediated sucrose biosynthesis is undoubtedly an important issue during cold stress (Almadanim *et al*., 2017; Strand *et al*., 2003). For example, *OsSPS4* was phosphorylated by OsCPK17 to accumulate sucrose concentration in rice, which is required for cold stress response (Almadanim *et al*., 2017). However, which upstream TFs regulate *SPS* expression under cold stress has not been reported either in the model or non‐model plants until now. Here, the Y1H, EMSA, LUC, and GUS transient expression assays consistently demonstrated that CdWRKY2 can bind to the *CdSPS1* promoter and activate its expression (Figure 5c–f). Moreover, overexpression of sucrose synthetic gene *CdSPS1* indeed improved cold tolerance in transgenic *Arabidopsis* (Figure 6a–d), while knockdown of *CdSPS1* expression improved susceptibility to cold stress in bermudagrass (Figure 6e–h), further revealing that CdWRKY2 confers cold resistance by directly activating *CdSPS1* expression to increase sucrose concentrations. Although the role of WRKY‐mediated sucrose synthesis in cold stress has not been reported yet, WRKY‐regulated sucrose synthesis has been documented to participate in other abiotic stresses, such as drought stress. For example, *VvWRKY30* improves drought tolerance partially by increasing the transcript level of the SS gene *SS4* (Zhu *et al*., 2018). Raineri *et al*. (2015) reported that *HaWRKY76*‐overexpressing *Arabidopsis* exhibits tolerance to flood and drought with higher sucrose contents relative to control.

In addition, sugar is an energy source that is important for photosynthesis. It was reported that the recovery of photosynthetic capacity under cold stress conditions is strongly dependent on the activation of the sucrose biosynthetic pathway (Strand *et al*., 2003). Herein, it is noteworthy that the PSII efficiency was rescued in *CdWRKY2*‐ or *CdSPS1*‐overexpressing *Arabidopsis* plants, but was suppressed more severely in *CdWRKY2*‐ or *CdSPS1*‐silencing bermudagrass plants during cold stress. Therefore, in addition to osmotic pressure, the photosynthetic ability is also improved by CdWRKY2‐mediated sucrose synthesis during cold stress of bermudagrass. Interestingly, there was no significant up‐regulation of sucrose content in *CdWRKY2‐*overexpressing hairy roots after cold stress although the *CdSPS1* expression was significantly induced in transgenic hairy roots (Figure 4g,j). Plants may activate negative feedback to avoid excess sucrose which may adversely affect plant growth and development, and its underlying mechanism is waiting to be explored.

It is worth noting that *CdSPS1* expression was only slightly activated by salt or drought stress (Figure S10) compared with cold stress. Therefore, the CdWRKY2‐CdSPS1 regulatory module is speculated to mainly play an important role in plants against cold stress rather than salt and drought stresses, although *CdWRKY2* expression levels were largely induced by salt and drought stresses.

### *CdCBF1* participates in CdWRKY2‐mediated cold stress response in bermudagrass

*CBFs* play a crucial role in cold stress, the expression of which was rapidly induced by cold temperature (Chinnusamy *et al*., 2007; Gilmour *et al*., 1998). Currently, more and more researches demonstrate that *CBF* expression is also regulated by multiple factors in addition to the most thoroughly understood ICE1‐CBF‐COR transcriptional cascade (Chinnusamy *et al*., 2007). For example, BZR1 positively regulates freezing tolerance via the CBF‐dependent pathway in *Arabidopsis* (Li *et al*., 2017), while VvWRKY34 might negatively mediate cold sensitivity through *CBF* signal cascade in *Arabidopsis* (Zou *et al*., 2010). However, there remain few reports on upstream regulators of CBFs‐dependent pathways in bermudagrass. In our study, *CdCBF1* expression was significantly increased about 100‐fold in *CdWRKY2‐*overexpressing hairy roots relative to the control, while its expression was dramatically down‐regulated in *CdWRKY2‐*silencing bermudagrass plants (Figures S4g and S5b). The interaction between CdWRKY2 and *CdCBF1* promoter was further validated by Y1H, EMSA, and LUC transient expression assays (Figure 7). Correspondingly, *COR,* which is the target gene of CBF showed the same expression pattern, indicating that the CdWRKY2 directly activates the *CdCBF1‐COR* signalling pathway during cold stress.

### Antioxidant genes may be involved in CdWRKY2‐mediated cold stress response in bermudagrass

It is well known that cold stress is always accompanied by the accumulation of ROS which stimulates membrane lipid peroxidation, resulting in cell membrane damage, even cell death (Ruelland *et al*., 2009). Plants have evolved a complex antioxidant system to cope with the oxidative injury induced by ROS (Miller *et al*., 2010). Increasing evidence suggests that antioxidant capacity is positively correlated with cold resistance (Hu *et al*., 2020; Sun *et al*., 2019). In this study, a class of genes that were co‐induced by cold stress and CdWRKY2 were noticeably enriched in response to the oxidative stress process, especially *UGT73B4* and *UGT74B2* (Figure 4d). *CsUGT78A14* has been reported to enhance cold tolerance by accumulating flavonol glycosides and improving ROS scavenging capacity (Zhao *et al*., 2019). Down‐regulating *UGT91Q2* reduced ROS scavenging capacity and accumulation of nerolidol glucoside, thus leading to lower cold tolerance in tea plants (Zhao *et al*., 2020). Correspondingly, the cell membrane stability was rescued by overexpressing *CdWRKY2* as indicated by decreased EL and MDA under cold conditions (Figure 2e,f). Therefore, the fine‐tuning of genes with antioxidant capacity may partially contribute to *CdWYKY2*‐mediated cold tolerance.

### *A*. *rhizogenes*‐mediated transformation system successfully removes barriers of functional analysis in bermudagrass

The genetic transformation with high efficiency is a determinative factor for investigating gene function and improving germplasm in bermudagrass. However, the *A. tumefaciens*‐mediated transformation system in bermudagrass has a very low transformation efficiency, which greatly hampers gene function analysis (Huang *et al*., 2018). For many plant species, a fast and efficient transformation technique with *A. rhizogenes* has been alternatively developed (Kereszt *et al*., 2007; Meng *et al*., 2019). In this study, *A. rhizogenes*‐mediated transformation has been successfully established in bermudagrass (Figure 3d–f). A series of hairy roots of bermudagrass with high expression of *CdWRKY2* were generated (Figure 3d,f). Although only transgenic hairy roots rather than the whole transgenic plant are obtained, *A. rhizogenes*‐mediated transformation has been well employed in investigation of abiotic stress previously. For example, soybean plants with *GmMYB118*‐overexpressing hairy roots generated via *A. rhizogenes*‐mediated transformation increased salt and drought tolerance (Du *et al*., 2018). The soybean seedlings with *WRKY54‐RNAi* hairy roots which are produced by *A. rhizogenes*‐mediated transformation show higher drought sensitivity (Wei *et al*., 2019). Here, the bermudagrass seedlings with *CdWRKY2‐*overexpressing hairy roots indeed enhanced cold tolerance.

**Figure 8:**
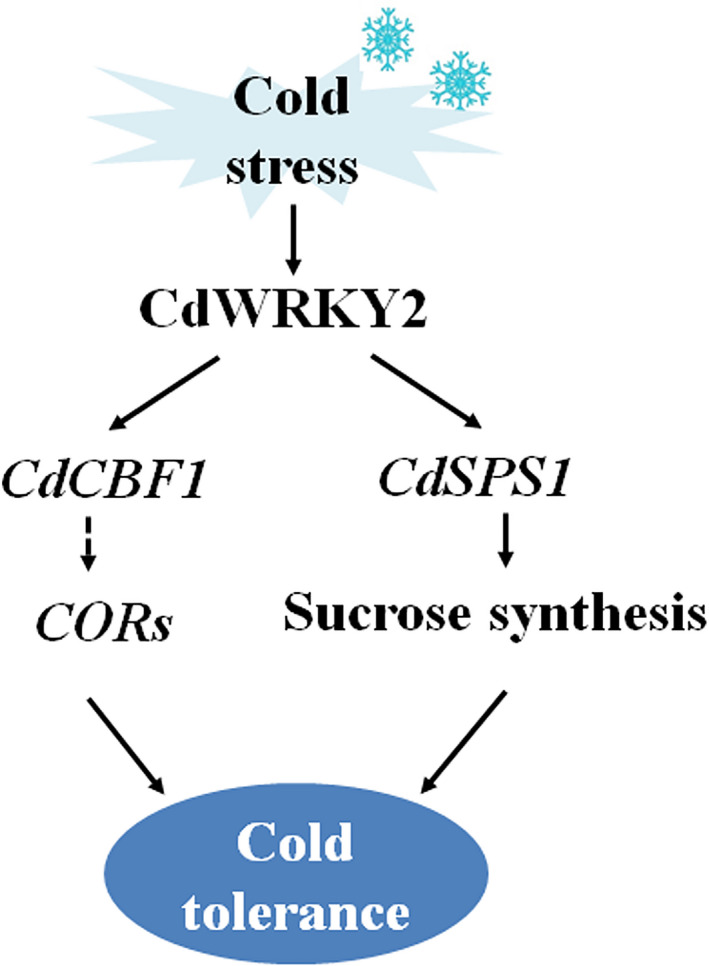
A proposed model for explaining the regulatory mechanism of CdWRKY2‐meidated cold stress response. *CdWRKY2* is significantly up‐regulated when bermudagrass is exposed to cold stress. On one hand, the cold‐induced CdWRKY2 directly activates *CdSPS1* expression to mediate sucrose biosynthesis, thus conferring cold tolerance of bermudagrass. On the other hand, CdWRKY2 directly binds to *CdCBF1* promoter to activate *CORs* expression, contributing to cold tolerance of bermudagrass.

In conclusion, our findings demonstrate that CdWYKY2 acts as a positive regulator in cold stress by coordinately activating *CdSPS1*‐involved sucrose synthesis and *CdCBF1*‐dependent signalling pathways (Figure 8). Moreover, we develop an efficient *A. rhizogenes*‐mediated transformation system in bermudagrass for gene functional analysis. The study provides valuable clues for the genetic modification of bermudagrass in response to cold stress.

## Experimental procedures

### Plant materials and growth conditions

In this study, the Columbia ecotype of *Arabidopsis thaliana* was used as the WT background. The plant seeds were sown on Petri dishes containing half‐strength Murashige and Skoog (MS) medium supplement with 3% sucrose and 0.8% agar after surface sterilisation and incubated at 4 °C for 2 days. Five‐day‐old seedlings were transferred to soil and moved to an artificial chamber under control environmental conditions (22 ± 1°C under a 16 h light/8 h dark cycle with a photon flux of 240 μmol/m/s).

Analogously, the cold‐resistant and cold‐sensitive bermudagrass (*C*. *dactylon*) genotypes screened previously were used here (Chen *et al*., 2015). Uniform stolons propagated from one original plant were planted in the plastic pots (7.5 cm in diameter and 9.0 cm deep) that were filled with nutrient soil (Beilei, Wuhan, China), then maintained in the artificial chamber with conditions of 12‐h‐light (240 μmol/m/s, 30 °C)/12‐h‐dark (28 °C) for about 2 months. During the establishing period, the plants were watered three times each week and fertilised weekly with full‐strength Hoagland’s solution.

### Cloning and sequence analysis

A 1683 bp CDS of *CdWRKY2* in cold‐resistant bermudagrass was amplified based on annotated transcriptome analysis described by Chen *et al*. (2015) using RACE kits (Takara, Dalian, China). The amino acid sequences of CdWRKY2 were used to search homologous proteins by the BLASTp program in the GenBank database (http://www.ncbi.nlm.nih.gov/). The phylogenetic tree was constructed using the MEGA6.0 software. Similarly, the  CDS sequence of *CdSPS1* was cloned according to the full‐length transcriptome of bermudagrass using reverse transcription PCR (Zhang et al., 2018b). The promoters of *CdWRKY2*, *CdSPS1*, and *CdCBF1* were amplified with specific primers (Table S1) using a genome walking kit (Takara, Dalian, China).

### Abiotic stresses treatments of bermudagrass

In order to investigate the changes of *CdWRKY2* and *CdSPS1* expression under cold stress, 2‐month‐old cold‐resistant and cold‐sensitive bermudagrass were transferred into the growth chamber with 4 °C for chilling treatment. For salt, drought, and ABA treatments, 2‐month‐old cold‐resistant bermudagrass were treated with 250 mM NaCl, 25% PEG6000, and 100 µM ABA, respectively. The leaf samples for qRT‐PCR analysis were harvested at 0, 3, 6, 12, and 24 h after treatments.

### RNA extraction and gene expression analysis

The total RNA from plants was extracted using Trizol‐reagent (Invitrogen, Carlsbad) and was digested by DNase I (Beyotime, Shanghai, China) before first‐strand cDNA synthesis. The first‐strand cDNA was obtained using the M‐MLV cDNA synthesis kit (Promega, Shanghai, China). qRT‐PCR was performed using a SYBR Green PCR Master Mix kit (Monad, Wuhan, China) on the Step One Plus Real‐Time PCR Systems (Applied Biosystems, Thermo Fisher Scientific Corp. 5). *AtACTIN2* and *CdACTIN2* were used as reference genes for *Arabidopsis* and bermudagrass, respectively. The relative expression levels were calculated as previously described (Hu *et al*., 2020). Primers used for qRT‐PCR are listed in Table S1.

### *Arabidopsis* transformation

To generate *CdWRKY2‐* and *CdSPS1‐*overexpressing transgenic plants, both *CdWRKY2* and *CdSPS1* CDS sequences were amplified from cold‐resistant bermudagrass and cloned into pMD35S vector. To generate *35S:CdWRKY2‐eGFP* transgenic plants, the *CdWRKY*2 CDS without the termination codon was amplified from cold‐resistant bermudagrass and inserted into pBI121‐eGFP vector. To generate *proCdWRKY2:GUS* transgenic plants, the *CdWRKY2* promoter region amplified from cold‐resistant bermudagrass was cloned into pCAMBIA1300‐GUS vector. Transgenic plants were obtained according to the floral dip method (Clough and Bent, 1998) and screened with 50 mg/L kanamycin or 25 mg/L hygromycin.

### Subcellular localisation of CdWRKY2

Seeds of the *35S:CdWRKY2‐eGFP* transgenic *Arabidopsis* were germinated on 1/2 MS medium after surface sterilisation, roots of five‐day‐old seedlings were used for fluorescence detection. The fluorescence signal of the 3*5S:CdWRKY2‐eGFP* fusion protein was observed using a DMI6000 CS confocal laser scanning microscope (Leica, Wetzlar, Germany).

### GUS staining

Five‐day‐old seedlings of *proCdWRKY2:GUS* transgenic *Arabidopsis* were treated with various abiotic stresses, including 4 °C (cold stress), 125 mM NaCl (salt stress), 25% PEG6000 (osmotic stress), and 100 μM ABA. After 6 h of various treatments, the seedlings were incubated in the X‐Gluc solution immediately for 2 h at 37 °C. Then, chlorophyll was removed using 70% (v/v) ethanol and the seedlings were photographed (Sun *et al*., 2019).

### *A*. *rhizogenes*‐mediated transformation system of bermudagrass

The seeds of commercial bermudagrass (Bailv Landscape Design Co., Ltd., Xi'an, China) were sown on 1/2 MS medium after surface sterilisation under dark conditions. After 2 weeks, the elongated stems were cut into 1 cm segments for *A. rhizogenes* infection. *A. rhizogene* strain MSU440 harbouring *35S:CdWRKY2‐eGFP* or empty vector (pBI121‐eGFP) was cultured in 50 mL LB liquid medium plus 20 mg/L streptomycin and 50 mg/L kanamycin until the OD_600_ value reached 0.5–0.6. The MSU440 suspension was centrifuged at 7656 *g* for 8 min under room temperature and then re‐suspended in a liquid infection medium (MS + 100 μM acetosyringone). The stem segments were incubated with *A. rhizogenes* suspension, vibrated in an ultrasonic cleaner for 5 min and then shake with 180 rpm at 28 °C for 0.5 h. Then, the infected stem segments were cultured on a co‐culture medium with (MS + 100 μM acetosyringone) at 22 °C under darkness. After 3 days, the segments were transferred into a screening medium (MS + 50 mg/L kanamycin + 300 mg/L cefotaxime) for about 1 month. Subsequently, the growing hairy roots were transferred into MS medium containing 50 mg/L kanamycin and subcultured for about 1 month. Finally, to exclude the potential contamination caused by *A. rhizogenes* as possible as we can, we chose the hairy roots without the outbreak of *A. rhizogenes* for the subsequent experiments.

### Virus‐induced gene silencing

To knock down the expression of *CdWRKY2* or *CdSPS1* in bermudagrass, the VIGS system was performed according to the method as previously described (Hu *et al*., 2020). Briefly, a specific fragment of *CdWRKY2* or *CdSPS1* was inserted into the pCa‐γbLIC vector under the control of CaMV35S promoter. The constructed vector was transformed into *A*. *tumefaciens* EHA105 which was then infiltrated into tobacco leaves. After 7 days, the infiltrated leaves were harvested and ground in 20 mM phosphate buffer (pH 7.2). Subsequently, the mixed liquid was inoculated into the leaves of 2‐month‐old bermudagrass via mechanical friction. Two weeks after infection, fully expanded leaves were collected from the transfected bermudagrass for qRT‐PCR to screen putative VIGS plants which were used for further analyses.

### Freezing treatments of *Arabidopsis* plants

For *Arabidopsis* seedlings on Petri dishes with CA, 10‐day‐old *Arabidopsis* seedlings were kept at 4 °C for 7 days, and then transferred into a freezing chamber with −7 °C for 1 h of freezing treatment. For *Arabidopsis* seedlings on Petri dishes with non‐acclimation (NA), 10‐day‐old seedlings were directly exposed to −6 °C for 1 h. After freezing treatments with or without CA, seedlings were transferred to 4 °C for 24 h, then recovered at 22 °C for an additional 3 days for phenotype observation and detection of survival rates.

For *Arabidopsis* plants in soil with CA, 3‐week‐old *Arabidopsis* plants were treated with 4 °C for 7 days, and then transferred into a freezing chamber with −9 °C for 8 h. For *Arabidopsis* plants in soil with NA, the age‐matched seedlings were directly transferred into 8 °C for 8 h of freezing treatment. The phenotype and survival rates were scored after 3 days of recovery at 22 °C.

### Measurement of physiological and histochemical staining

For physiological analyses, both *Arabidopsis* and bermudagrass plants were treated at 4 °C for 7 days. The relative EL and MDA contents were measured as previously described (Hu *et al*., 2020). The chlorophyll fluorescence transient curve was recorded by pulse‐amplitude modulation (PAM) fluorimeter (PAM2500, Walz, Germany) and the OJIP curves were analysed using the JIP‐test method described by Yusuf *et al*. (2010). Histochemical staining of O_2_^−^ was conducted with NBT as previously reported (Hu *et al*., 2020). O_2_^−^ content was measured by using the detection kit (Solarbio, Beijing, China) according to manufacturer’s instruction. In brief, about 0.1 g roots were ground and homogenised in extracting solution, then centrifuged at 4 °C with 13 800 *g* for 20 min. The supernatant was collected for further analysis. The absorbance of the supernatant at 530 nm was measured using a microplate reader (M200 PRO, TECAN, Männedorf, Switzerland).

Sucrose contents were detected using Sucrose Assay Kit (Solarbio, Beijing, China) according to the instruction. Briefly, about 0.1 g leaves were ground and homogenised in extracting solution. The mixed solution was incubated at 80 °C for 10 min in the water bath, then fast‐cooled to room temperature and centrifuged at 25 °C with 1500 *g* for 10 min. The supernatant was decolourised using activated carbon at 80 °C for 10 min, and then added into 1 mL extracting solution. The mixed solution was centrifuged at 25 °C with 1500 *g* for 10 min and the supernatant was collected for further analysis. The absorbance of the supernatant at 408 nm was measured using a microplate reader (M200 PRO, TECAN, Männedorf, Switzerland).

### RNA‐Seq analysis

RNA‐Seq analysis was performed using 7‐day‐old seedlings of WT and *CdWRKY2‐*overexpressing transgenic *Arabidopsis* before or after 6 h of 4 °C treatments, named WTCK, OE3CK, WTLT, and OE3LT, respectively. Total RNA was extracted using the Trizol kit (Invitrogen, Carlsbad, CA) from seedlings. Two biological replicates were used for each sample, and the RNA quality and concentration were detected by NanoDrop 2000 (Thermo, Waltham) and a 1.2% agarose gel. The cDNA libraries were constructed and sequenced in Novogene Company (Beijing, China) according to the standard procedure. The raw data were filtered to remove the low‐quality reads (Q value ≤ 20) and then the clean date was mapped to the *Arabidopsis thaliana* genome. The gene expression levels of each sample were calculated by FPKM method. The DEGs were analysed using DESeq2 software. GO analysis and KEGG classifications by DEGs were performed using clusterProfile software.

### Yeast one‐hybrid assay

For Matchmaker Gold Y1H Library Screening System, the CDS of *CdWRKY2* was fused to the GAL4 AD in the pGADT7 vector to generate the prey vector (pGADT7‐CdWRKY2), while the promoters of *AtSUS1* or *AtSPS2F* was inserted into the pAbAi vector to construct the baits (*proAtSUS1/AtSPS2F‐AbAi*). The pGADT7‐CdWRKY2 vector was transformed into the Y1H Gold yeast strain. After selecting the transformants on SD/−Ura plates, BstBI‐cut bait vector was introduced into the Y1H Gold yeast strain containing pGADT7‐CdWRKY2. Positively co‐transformed cells were screened on SD/−Leu medium supplemented with AbA and cultured at 30 °C for 3 days. A positive (pGADT7‐p53 + *p53‐AbAi*) control was processed in the same manner.

For the EGY48 Y1H system, the CDS regions of *CdWRKY2* were fused to the GAL1 AD in the pB42AD vector to generate the prey vector (pB42AD‐CdWRKY2), while the promoter of *CdSPS1* and *CdCBF1* was fused to the pLacZi vector to construct the baits. pB42AD‐CdWRKY2 vector was transformed into the EGY48 yeast strain. After selecting the transformants on SD/−Trp plates, the NcoI‐cut bait vector was introduced into the EGY48 yeast strain harbouring pB42AD‐CdWRKY2. Positively co‐transformed cells were screened on SD/−Trp/‐Ura medium and cultured at 30 °C for 3 days. The resultant transformants were tested for β‐galactosidase activity on selective media (SD/−Trp/‐Ura/BU salt/X‐gal). A positive (pB42AD‐p53 + *p53‐LacZi*) control was processed in the same manner.

### Electrophoretic mobility shift assay

The CDS of *CdWRKY2* was cloned into pGEX‐6p‐1 entry vector, and then were transformed into *Escherichia coli* BL21 (DE3) competent cells. The GST‐WRKY2 fusion protein was induced by 1 mM Isopropyl‐β‐d‐thiogalactopyranoside at 30 °C for 4 h and purified using the GSTSep Glutathione Agarose Resin (Yeasen, Shanghai, China) according to the manufacturer’s instructions. The purified GST‐CdWRKY2 fusion protein and the biotin‐labelled DNA probes containing either WT or mutated W‐box (Table S1) were used for EMSA. The EMSA was carried out using the Light Shift Chemiluminescent EMSA Kit (Beyotime, Shanghai, China) according to the manufacturer’s instructions. Photos were obtained by a multifunctional imaging system (FluorChem R, Proteinsimple, America).

### LUC reporter assay

For transient expression assays, the promoter region of *CdSPS1* and *CdCBF1* were ligated into pGreenII‐0080‐LUC to generate the reporter construct, *proCdSPS1‐LUC* and *proCdCBF1‐LUC*, respectively. The *35S:CdWRKY2* construct was served as an effector. The effector and reporter constructs were transformed into *A. tumefaciens* strain GV3101 harbouring pSoup helper vector, respectively, which were further co‐infected *Nicotiana benthamiana* leaves. The injected tobacco plants were kept in the dark for 2 days and then 1 days in the normal condition. Transient expression was reflected by measuring firefly LUC and REN luciferase activities using the Dual‐Luciferase Reporter Assay System according to the manufacturer’s instructions (Promega, Wisconsin). Five biological replications were measured for each sample.

### GUS reporter assay

The same 900‐bp promoter region of *CdSPS1* was ligated into pCAMBIA1300‐GUS to generate the reporter construct, *proCdSPS1‐GUS*. The *35S:CdWRKY2* construct was used as an effector. The effector and reporter constructs were transformed into *A. tumefaciens* strain GV3101, and then were co‐infected into *N. benthamiana* leaves. The GUS activity was assayed according to a previous method (Zhang *et al*., 2020). Five biological replications were measured for each sample.

### Statistical analysis

All experiments in this study were performed with at least three repetitions. The significance of differences was determined by ANOVA or Student’s *t*‐test using IBM SPSS 20 software, as indicated in the figure legends.

## Conflict of interest

The authors declare that they have no competing interests.

## Author contributions

LC designed the research; XBH, JBF, and GJM performed experiments; XBH and LWC analysed data; XBH drafted the manuscript; LC and LWC revised the manuscript. XBH and LWC contributed equally.

## Supporting information


**Figure S1** Characteristic analysis of CdWRKY2.
**Figure S2** The differences of cis‐acting elements in *CdWRKY2* promoter region between cold‐sensitive (S) and cold‐resistance (R) bermudagrass genotypes.
**Figure S3** *CdWRKY2* expression and photosynthesis indexes in *CdWRKY2*‐overexpressing *Arabidopsis* plants.
**Figure S4** Silencing of *CdWRKY2* by VIGS leads to impaired cold tolerance in bermudagrass.
**Figure S5** Expression patterns of cold marker genes in *HREV* and *HROEs* after cold treatment.
**Figure S6** The KEGG pathway analyses.
**Figure S7** GO analyses.
**Figure S8** *CdSPS1* expression and photosynthesis indexes in *CdSPS1‐*overexpressing *Arabidopsis* plants.
**Figure S9** Silencing of *CdSPS1* by VIGS leads to impaired cold tolerance in bermudagrass.
**Figure S10** Expression patterns of *CdSPS1* under abiotic stresses.
**Table S1** Primers used in the study.
