# Supplementary material for: CdWRKY2‐mediated sucrose biosynthesis and CBF‐signalling pathways coordinately contribute to cold tolerance in bermudagrass
Source: Plant Biotechnol J. 2021 Nov 23;20(4):660–75. doi: 10.1111/pbi.13745 (PMC8989505; doi:10.1111/pbi.13745)
Supplement: Supplementary file 1 — Figure S1 Characteristic analysis of CdWRKY2. Figure S2 The differences of cis‐acting elements in CdWRKY2 promoter region between cold‐sensitive (S) and cold‐resistance (R) bermudagrass genotypes. Figure S3 CdWRKY2 expression and photosynthesis indexes in CdWRKY2‐overexpressing Arabidopsis plants. Figure S4 Silencing of CdWRKY2 by VIGS leads to impaired cold tolerance in bermudagrass. Figure S5 Expression patterns of cold marker genes in HREV and HROEs after cold treatment. Figure S6 The KEGG pathway analyses. Figure S7 GO analyses. Figure S8 CdSPS1 expression and photosynthesis indexes in CdSPS1‐overexpressing Arabidopsis plants. Figure S9 Silencing of CdSPS1 by VIGS leads to impaired cold tolerance in bermudagrass. Figure S10 Expression patterns of CdSPS1 under abiotic stresses. Table S1 Primers used in the study. [file PBI-20-660-s001.pdf]

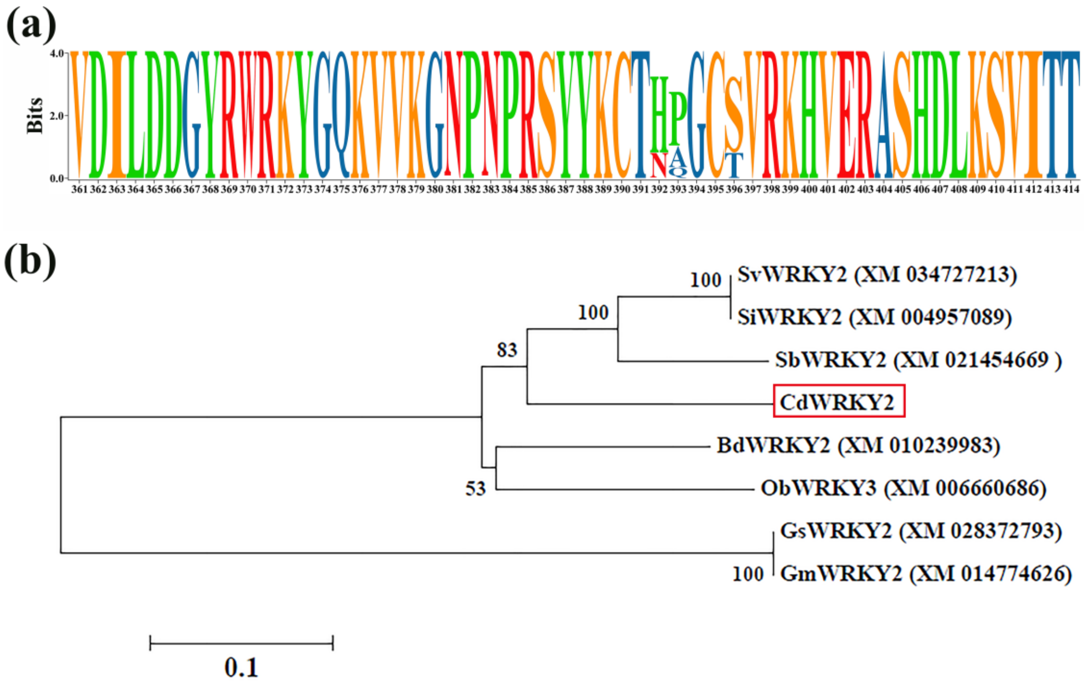

Figure S1 Characteristic analysis of CdWRKY2. (a) The conserved domain analysis of CdWRKY2. **(b)** The phylogenetic relationship between CdWRKY2 and other WRKY proteins, including SvWRKY2 (XM_034727213) from *Setaria viridis*, SiWRKY2 (XM_004957089) from *Setaria italic*, SbWRKY2 (XM_021454669) from *Sorghum bicolor*, BdWRKY2 (XM_010239983) from *Brachypodium distachyon*, ObWRKY2 (XM_006660686) from *Oryza brachyantha*, GsWRKY2 (XM_028372793) from *Glycine soja*, and GmWRKY2 (XM_014774626) from *Glycine max*. GenBank accession numbers of the proteins are listed in the brackets.

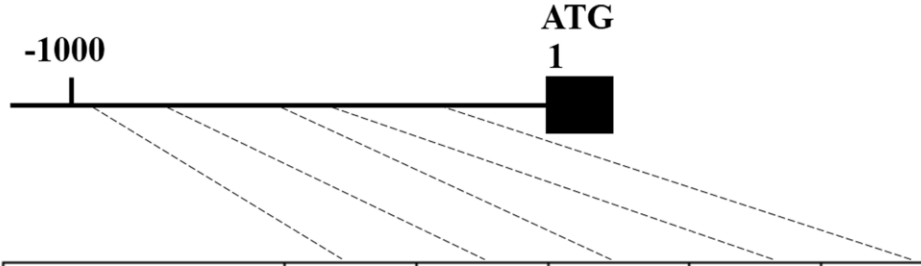

Variety	-867	-771	-484	-389	-250
R	*	C	G	*	C
S	C	T	A	T	T

Figure S2 The cis-acting element differences in *CdWRKY2* promoter region between R and S bermudagrass genotypes. Line (-) indicates base deletion.

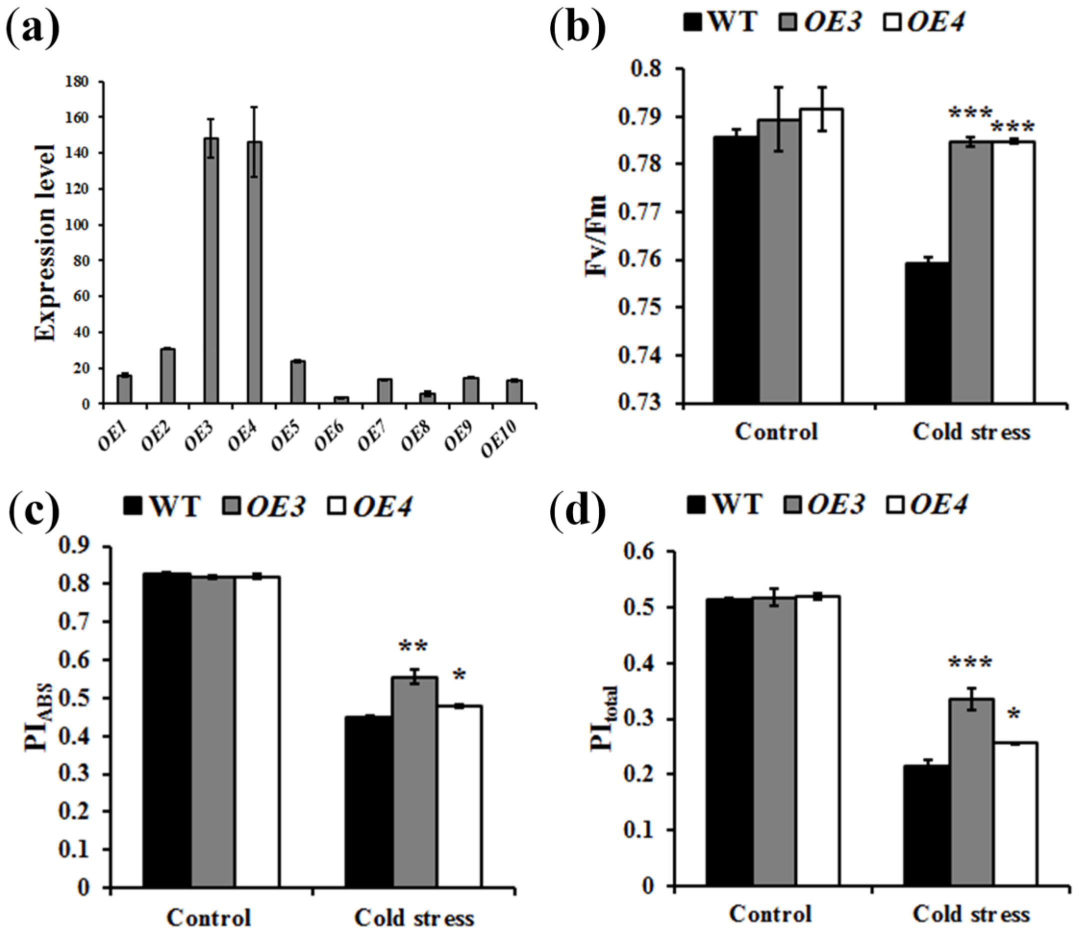

Figure S3 *CdWRKY2* expression and photosynthesis indexes in *CdWRKY2*-overexpressing *Arabidopsis* plants. (a) The expression level of *CdWRKY2* in transgenic *Arabidopsis* lines. **(b-d)** Fv/Fm ratios **(b)**, PI_{ABS} **(c)**, and PI_{total} **(d)** values in WT and two transgenic *Arabidopsis* lines under normal and cold stress (4°C for 7 d). The error bars indicate the SD values from at least three repeats of each treatment. Asterisks indicate significant differences (*$P < 0.05$; *$P < 0.01$; ***$P < 0.001$) between the transgenic lines and WT under the same growth conditions (Student's *t*-test).

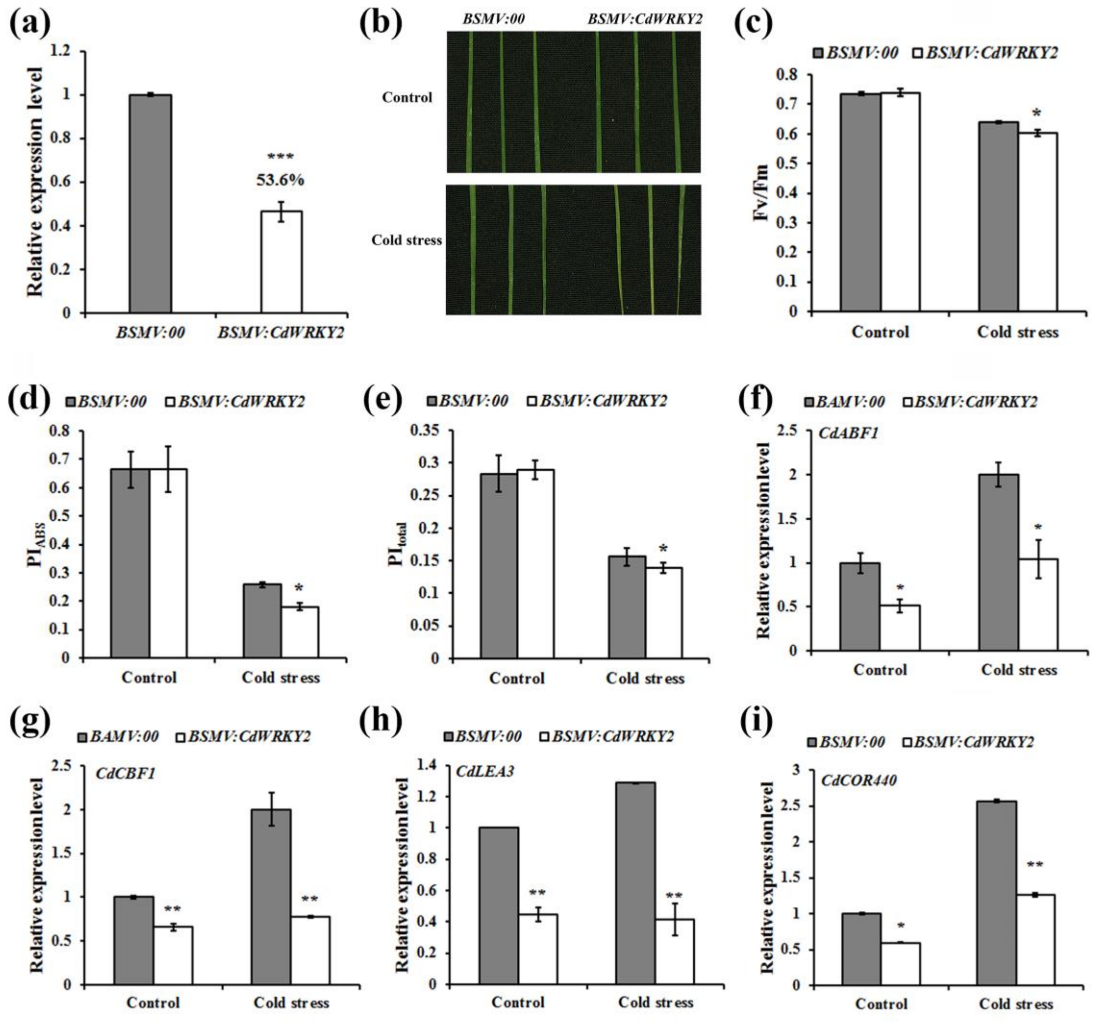

Figure S4 Silencing of *CdWRKY2* by virus-induced gene silencing (VIGS) leads to impaired cold tolerance in bermudagrass. (a) Relative expression levels of *CdWRKY2* in *BSMV:00* and *BSMV:CdWRKY2*. **(b)** Phenotypes of *BSMV:00* and *BSMV:CdWRKY2* bermudagrass under normal and cold stress conditions (4°C for 3 weeks). **(c-e)** Fv/Fm ratios **(c)**, PI_{ABS} **(d)**, and PI_{total} **(e)** values in *BSMV:00* and *BSMV:CdWRKY2* under normal and cold stress (4°C for 7 d). **(f-i)** Expression levels of cold marker genes including *CdABF1* **(f)**, *CdCBF1* **(g)**, *CdLEA3* **(h)**, and *CdCOR440* **(i)** in *BSMV:00* and *BSMV:CdWRKY2* before and after 6 h of 4°C treatment. *CdACTIN2* was used as normalization controls for qRT-PCR. The error bars indicate the SD values from at least three repeats of each treatment. Asterisks indicate significant differences (*P < 0.05; **P < 0.01; ***P < 0.001) between *BSMV:00* and *BSMV:CdWRKY2* under the same growth conditions based on Student's *t*-test.

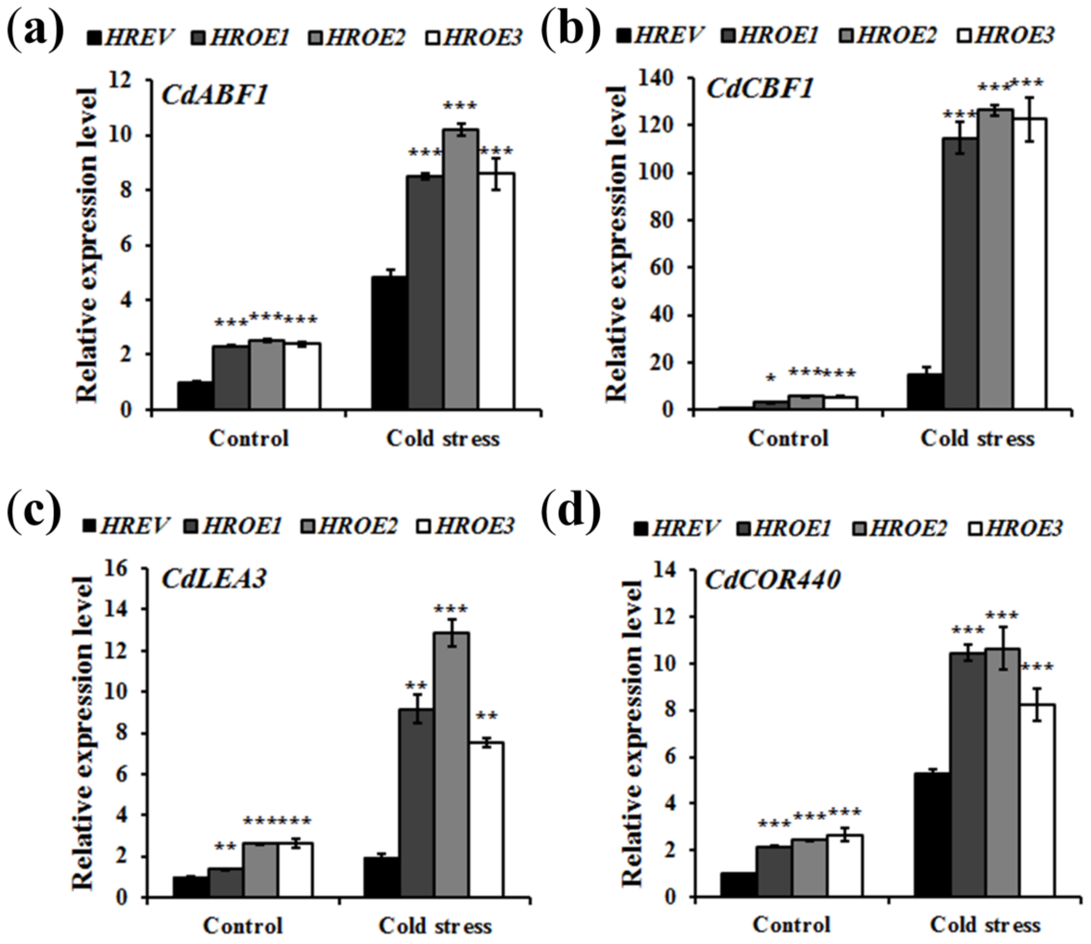

Figure S5 Expression patterns of cold marker genes in *HREV* and *HROEs* after cold treatment. (a-d) Expression analyses of *CdABF1* **(a)**, *CdCBF1* **(b)**, *CdLEA3* **(c)**, and *CdCOR440* **(d) i**n *HREV* and *HROEs* after 6 h of 4°C treatment**.** The error bars indicate the SD values of each treatment (n=3). Asterisks indicate significant differences (*P<0.05; **P < 0.01; ***P < 0.001) between *HREVs* and *HROEs* under the same growth conditions.

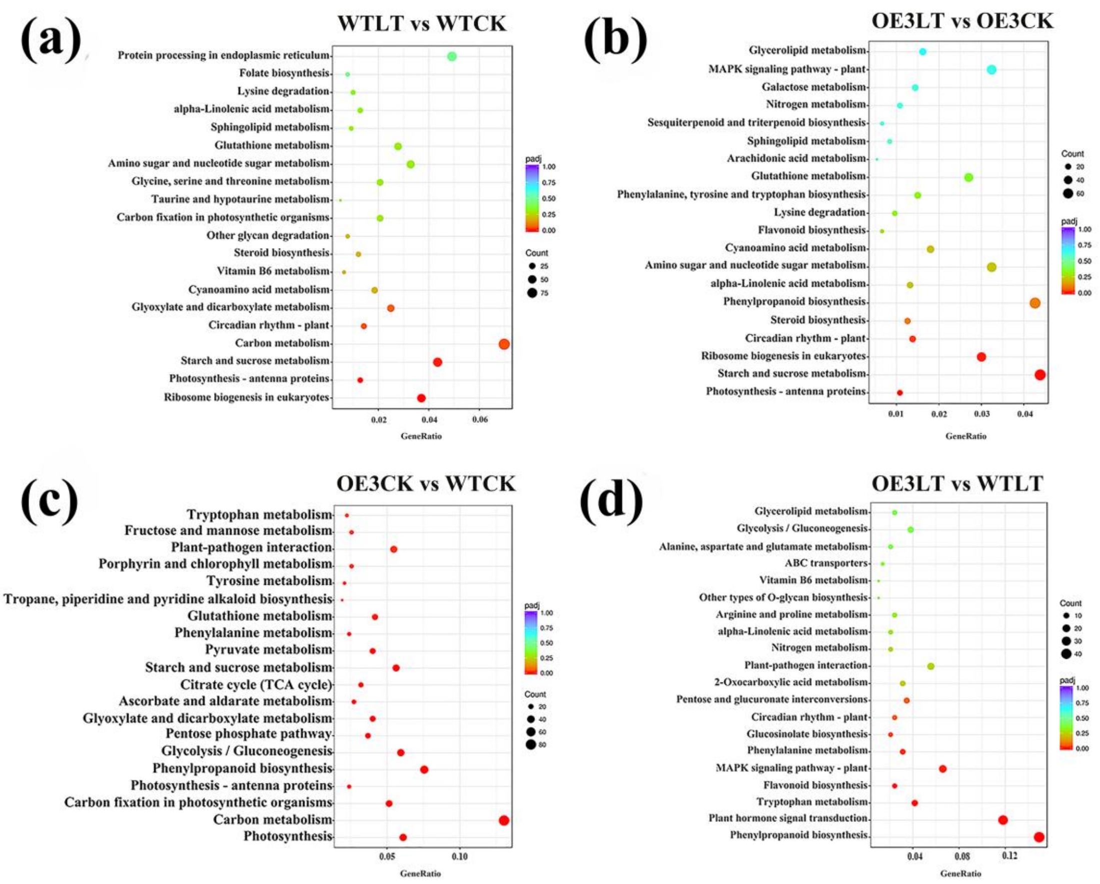

Figure S6 The KEGG pathway analyses. The KEGG pathways of the differentially expressed genes (DEGs) in four comparisons including WTLT vs WTCK **(a)**, OE3LT vs OE3CK **(b)**, OE3CK vs WTCK **(c)**, and OE3LT vs WTLT **(d)**.

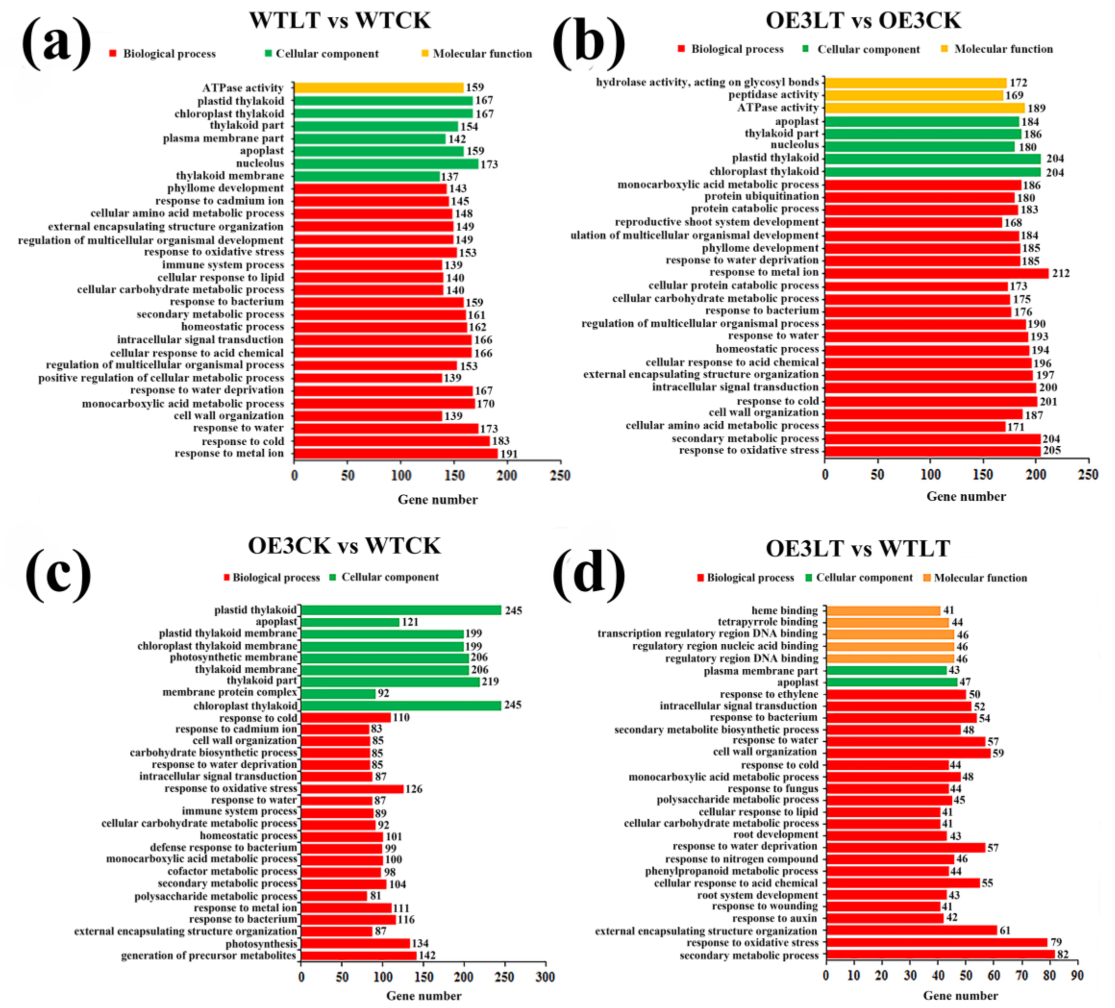

Figure S7 GO analyses. GO analysis of DEGs in four comparisons including WTLT vs WTCK **(a)**, OE3LT vs OE3CK **(b)**, OE3CK vs WTCK **(c)**, and OE3LT vs WTLT **(d)**.

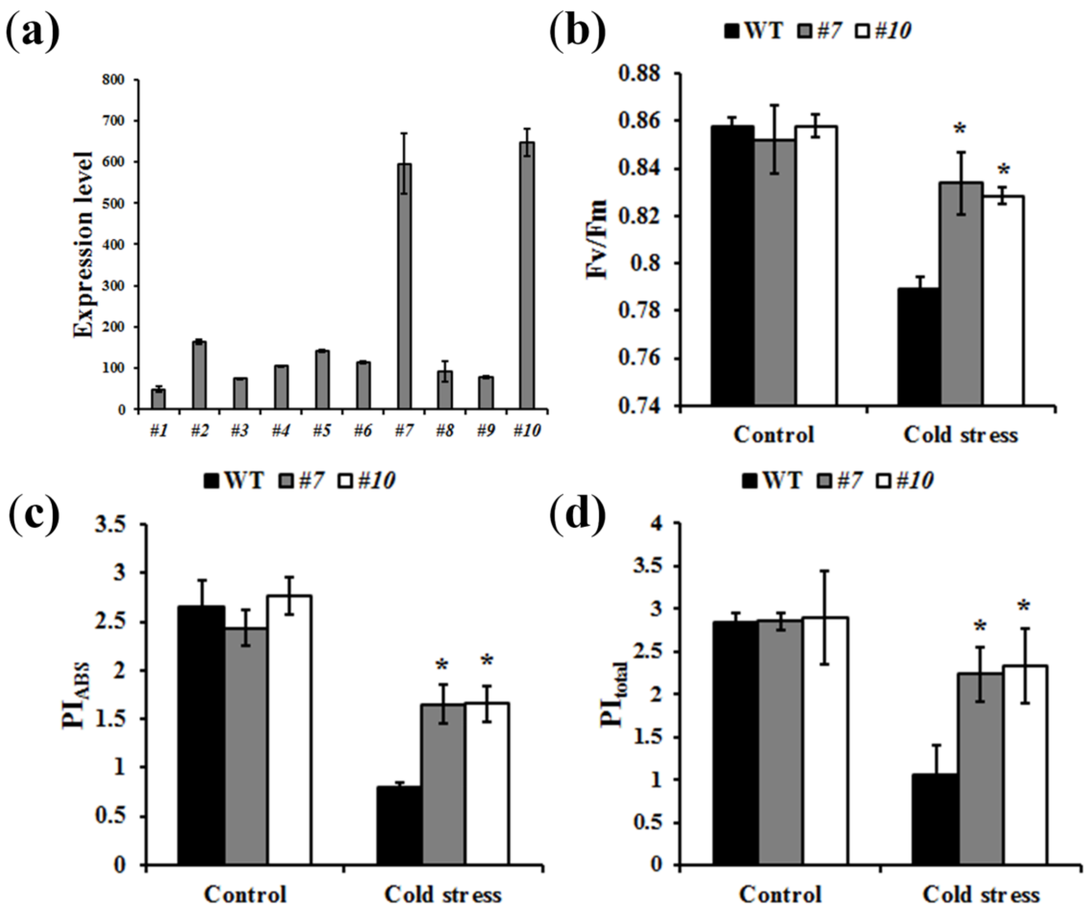

Figure S8 ***CdSPS1*** **expression and photosynthesis indexes in** ***CdSPS1*****-overexpressing** ***Arabidopsis*** **plants. (a)** The expression level of *CdSPS1* in transgenic *Arabidopsis* lines. **(b-d)** Fv/Fm ratios **(b),** PI_{ABS} **(c)**, and PI_{total} **(d)** values in WT and two transgenic *Arabidopsis* lines under normal and cold stress (4°C for 7 d). The error bars indicate the SD values from at least three repeats of each treatment. Asterisks indicate significant differences (*$P < 0.05$; ***$P < 0.001$) between the transgenic lines and WT under the same growth conditions (Student's *t*-test).

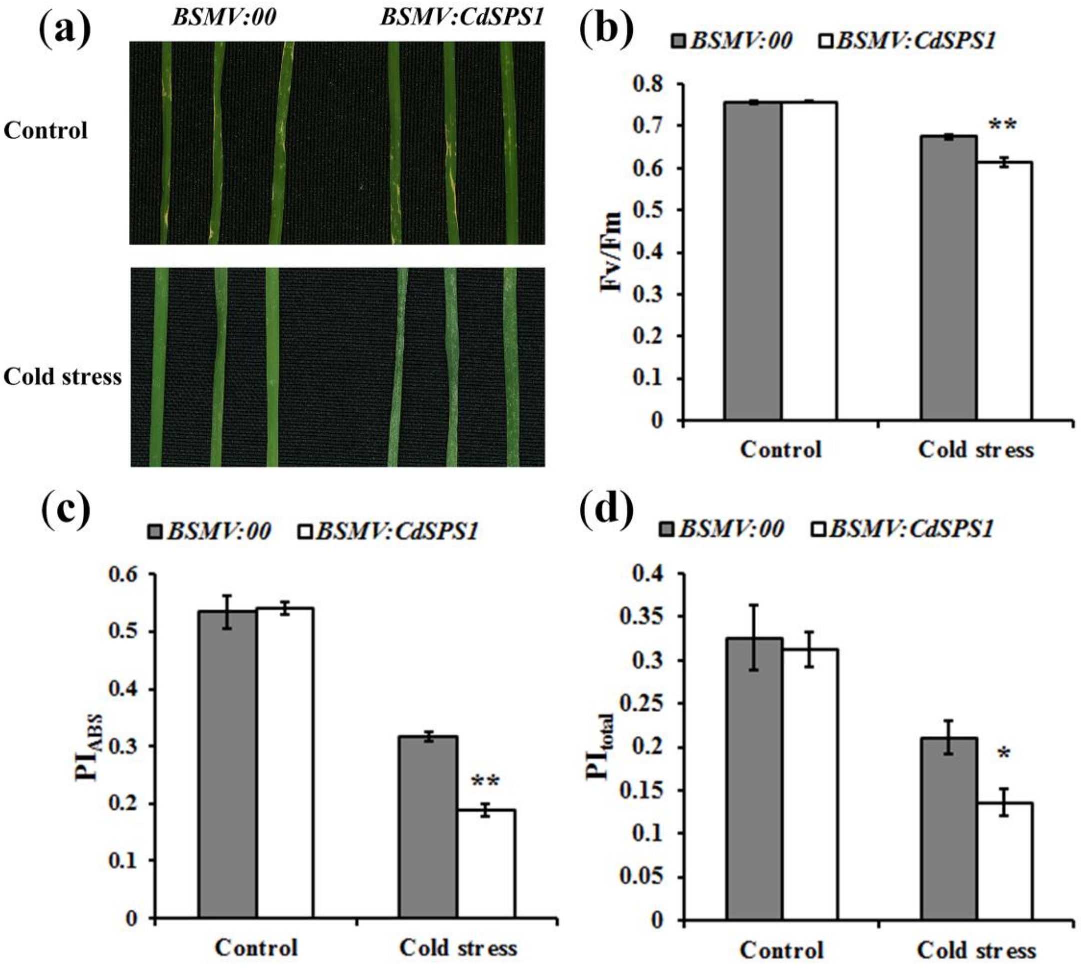

Figure S9 Silencing of *CdSPS1* by VIGS leads to impaired cold tolerance in bermudagrass. (a) Phenotypes of *BSMV:00* and *BSMV:CdSPS1* bermudagrass under normal and cold stress conditions (4°C for 3 weeks). **(b-d)** Fv/Fm ratios **(b)**, PI_{ABS} **(c)**, and PI_{total} **(d)** values in *BSMV:00* and *BSMV:CdSPS1* under normal and cold stress (4°C for 7 d). The error bars indicate the SD values from at least three repeats of each treatment. Asterisks indicate significant differences (*P < 0.05; **P < 0.01) between *BSMV:00* and *BSMV:CdSPS1* under the same growth conditions based on Student's *t*-test.

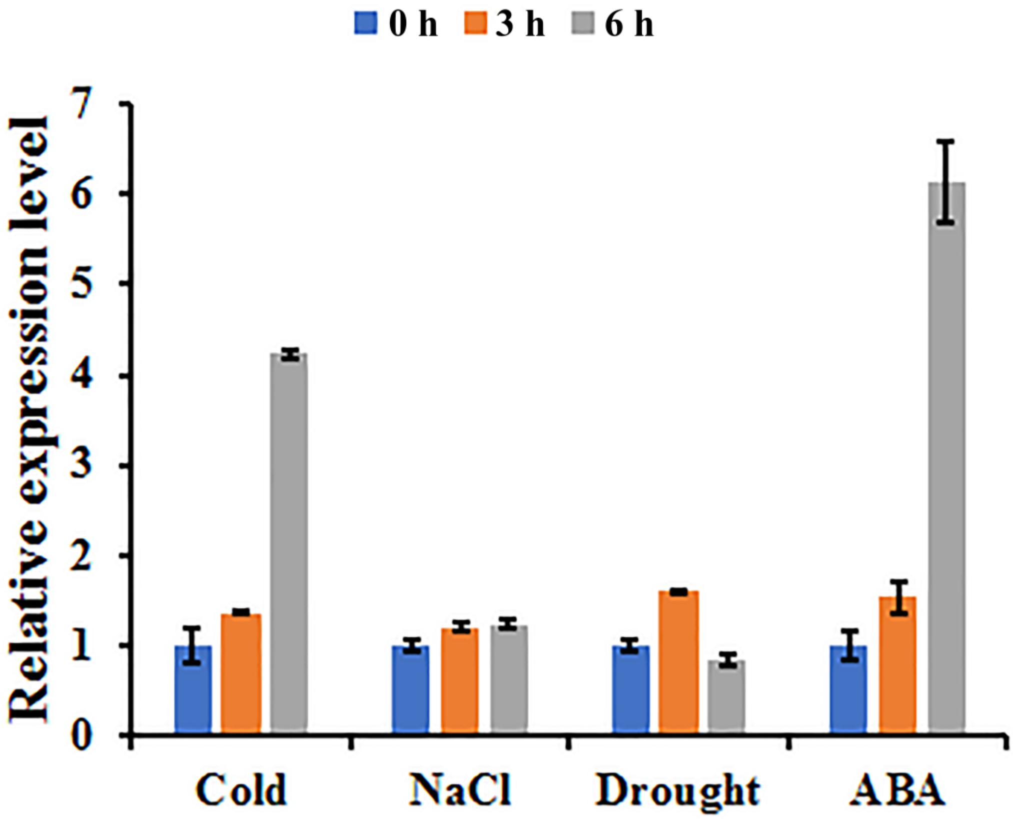

Figure S10 Expression patterns of *CdSPS1* under abiotic stresses. Time-course changes in expression levels of *CdSPS1* in response to 4°C cold stress, 200 mM NaCl, 25% PEG6000, and 100 μM ABA in cold-resistant bermudagrass. The error bars indicate the SD values (n=3). The different letters indicate significant statistical differences of *CdSPS1* expression among different treatment time under the same treatment conditions, respectively (Duncan's multiple range tests at $P < 0.05$).

Table S1 All primers used in this study

Gene	Primer sequences	Gene	Primer sequences
CdWRKY2-RT-F	AGCCTCGGGTTGTTGTTCA	CdWRKY2Pro-SP1	GAATATTGAAACCTGCTCTCGCC
CdWRKY2-RT-R	GTGCTTCCGCACTGAACAA	CdWRKY2Pro-SP1	GAGAAGCTTGAGCAGACATCTC
CdWRKY2-VIGS-F	AAGGAAGTTTAAGCAGCACAAGCGTCTCA	CdWRKY2Pro-SP1	GAGATAGGGCCAGAGCTGAATTCT
CdWRKY2-VIGS-R	AACCACCACCACCGTCATTTGAGGACCAAAAGGA	pCdWRKY2 GUS F	GTAgtcgacGCATAGGGCATACTGTGTTC
CdWRKY2-OE-F	ATATCTAGAATGGCCGGCACAAGTGACCA	pCdWRKY2 GUS R	GTAggatccGCAGCAAGAAATCCATCTCT
CdWRKY2-OE-R	GCGGTCGACTTACATTTGAGGACCAAAAG	CdACTIN2-F	TCTGAAGGGTAAGTAGAGTAG
CdWRKY2-eGFP-F	ATATCTAGAATGGCCGGCACAAGTGACCA	CdACTIN2-R	ACTCAGCACATTCCAGCAGAT
CdWRKY2-eGFP-R	GCGTCTAGACATTTGAGGACCAAAAGGAG	CdCOR440-RT-F	ATCAAGGAGAAGCTCCCGGGC
CdWRKY2-pB42AD-F	ATAGAATTCATGGCCGGCACAAGTGACCA	CdCOR440-RT-R	AGAGCTGGTCTTGTGCTCGCC
CdWRKY2-pB42AD-R	GCGGAATTCTTACATTTGAGGACCAAAAGG	CdLEA3-RT-F	TCATCCCCAGCGTGTTCATCA
CdWRKY2-pGADT7-F	ATACATATG ATGGCCGGCACAAGTGACCA	CdLEA3 -RT-R	GAGGCCGCCAAACAGAAGACA
CdWRKY2- pGADT7-R	GCGGAGCTCTTACATTTGAGGACCAAAAG	CdABF1-RT-F	AATGGATTGGTGACGGGAG
CdSPS1Pro-SP1	GATCAGCAGCAGCATATTGACG	CdABF1-RT-R	CATTGAAAACGTATGGCACTGG
CdSPS1Pro-SP2	TAAAGGTCGGTCTCGTCGAAGC	CdCBF1-RT-F	ACCAAGTTCCGCGAGACGC
CdSPS1Pro-SP3	ATGGCCTCCAGGTAACTGTTGA	CdCBF1-RT-R	CGAGTCGGCGAAGTTGAGGCA
proCdSPS1 pLacZ F	GTACggtaccATGTACACGTCAGCACGGAG	AtSPS2F-RT-F	CAACAGAAGCTACCCAATGG
proCdSPS1 pLacZ R	TATAggatccTCTCTCCGCGACGAGAGATT	AtSPS2F-RT-R	AGTCCTCAAGGCTTGAGGAGA
proCdSPS1 GUS F	TATAgtcgacATGTACACGTCAGCACGGAG	AtSUS1-RT-F	CTGGAAGCATGTCTCGAACCT
proCdSPS1 GUS R	TATAggatccTCTCTCCGCGACGAGAGATT	AtSUS1-RT-R	AACACCGGAACCACTCTCTTC
proCdSPS1 LUC F	GTACaagcttATGTACACGTCAGCACGGAG	AtACTIN2-F	GAAATCACAGCACTTGCACC
proCdSPS1 LUC R	TATAggatccTCTCTCCGCGACGAGAGATT	AtACTIN2-R	AAGCCTTTGATCTTGAGAGC
CdSPS1 RT F	AGATGTCCAAGGTGCTTGGT	AtSPS2F-pAbAi F	GCGCGAGCTCTCATTCTATCTTCTACTC
CdSPS1 RT R	TGTGAATACCATGGGCACGT	AtSPS2F-pAbAi R	TATACTCGAGTGGTGTTCGCTCTCCCGAAC
CdSPS1 OE F	GATATCTAGAATGGCGGGGAACGACTGGAT	AtSUS1-pAbAi F	GAGTGAGCTCCCAAAGATCAGTGACATATCCG

CdSPS1 OE R	GCGAGTCGACCTAGAGAATGACCTGTGAAT	AtSUS1-pAbAi R	GCTGTCGACTGATCCAAAAAAGAGACGCAG
CdSPS1-VIGS F	AAGGAAGTTTAACGGGGAACCTACTGAGATG	AtSPS2F-LUC F	GCGGTCGACTCATTCTATCTTCTACTC
CdSPS1-VIGS R	AACCACCACCACCGTGAGAATGACCTGTGAATACC	AtSPS2F-LUC R	TATAGGATCCTGGTGTTCGCTCTCCCGAAC
CdWRKY2-3'RACE Outer primer	AGCTGCTTATTGCCAGGATCACTG	CdWRKY2-3'RACE Inner primer	GCTCTGCCAAACCACCGTGTGAGT
35S F	CCACGTCTTCAAAGCAAGTGGATTGAT GTG	CdSPS1 Probe F	ATTCATCGCGAGCCAGCCTGACATACCTTTAGAAAAAATG
CdCBF Probe F	AGTCATGGTTAAAGTGCTTGACCGATCCAATCTGTTCTCT	CdSPS1 Probe R	CATTTTTTCTAAAGGTATGTCAGGCTGGCTCGCGATGAAT
CdCBF Probe R	AGAGAACAGATTGGATCGGTCAAGCACTTTAACCATGACT	CdSPS1m Probe F	ATTCATCGCGAGCCAGCCTTGCATACCTTTAGAAAAAATG
CdCBFm Probe F	AGTCATGGTTAAAGTGCTTTGCCGATCCAATCTGTTCTCT	CdSPS1m Probe R	CATTTTTTCTAAAGGTATGCAAGGCTGGCTCGCGATGAAT
CdCBFm Probe R	AGAGAACAGATTGGATCGGCAAAGCACTTTAACCATGACT		

F and R represent forward and reverse, respectively.
